# Bidirectional Associations Between Adiposity and Cognitive Function and Mediation by Brain Morphology in the ABCD Study

**DOI:** 10.1001/jamanetworkopen.2022.55631

**Published:** 2023-02-16

**Authors:** Mohammad Nazmus Sakib, John R. Best, Peter A. Hall

**Affiliations:** 1 School of Public Health Sciences, Faculty of Health, University of Waterloo, Waterloo, Ontario, Canada; 2 Gerontology Research Centre, Simon Fraser University, Burnaby, British Columbia, Canada; 3 Centre for Bioengineering and Biotechnology, University of Waterloo, Waterloo, Ontario, Canada; 4 Department of Psychology, University of Waterloo, Waterloo, Ontario, Canada

## Abstract

**Importance:**

Most epidemiologic studies examine the brain as an outcome in relation to adiposity (ie, the brain-as-outcome perspective), but it is also a potential risk factor associated with adiposity accumulation over time (ie, the brain-as–risk factor perspective). The bidirectionality hypothesis has not been fully explored in adolescent samples previously.

**Objective:**

To assess bidirectional associations between adiposity and cognitive function in youth and test mediational pathways through brain morphology (specifically the lateral prefrontal cortex [LPFC]), lifestyle behaviors, and blood pressure.

**Design, Setting, and Participants:**

This cohort study uses data (wave 1-3; 2 years of follow-up) from the Adolescent Brain Cognitive Development (ABCD) Study, a long-term longitudinal investigation of brain development in the United States launched in 2015, which recruited 11 878 children aged 9 to 10 years at inception. Data analysis was performed from August 2021 to June 2022.

**Main Outcomes and Measures:**

Multivariate multivariable regression analyses were used to assess bidirectional associations of indicators of cognitive function (eg, executive function, processing speed, episodic memory, receptive vocabulary and reading skills) and adiposity (eg, body mass index z scores [zBMI] and waist circumference [WC]). Mediators considered for this investigation were lifestyle variables (eg, diet and physical activity), blood pressure, and the morphology of the LPFC and its subregions.

**Results:**

A total of 11 103 individuals (mean [SD] age, 9.91 [0.6] years; 5307 females [48%]; 8293 White individuals [75%] and 2264 Hispanic individuals [21%]) were included in the current study. Multivariate multivariable regression analyses revealed that higher baseline zBMI and WC were associated with worse follow-up episodic memory (*β,* −0.04; 95% CI, −0.07 to −0.01) and better vocabulary (*β*, 0.03; 95% CI, 0.002 to 0.06) task performance, in covariate adjusted models. Similarly, superior baseline executive function (zBMI: *β,* −0.03; 95% CI, −0.06 to −0.01; WC: *β,* −0.04; 95% CI, −0.07 to −0.01) and episodic memory (zBMI: *β,* −0.04; 95% CI, −0.07 to −0.02; WC: *β,* −0.03; 95% CI, −0.06 to −0.002) task performance were associated with better follow-up adiposity status in covariate adjusted models. Cross-lagged panel models with latent variable modeling had a bidirectional association with executive function task performance (brain-as-outcome: *β,* −0.02; 95% CI, −0.05 to −0.001; brain-as–risk factor: *β,* −0.01; 95% CI, −0.02 to −0.003). The hypothesized associations were statistically mediated by LPFC volume and thickness, physical activity, and blood pressure.

**Conclusions and Relevance:**

In this cohort study, executive function and episodic memory were bidirectionally associated with adiposity indices over time in this adolescent sample. These findings suggest that the brain can be both a risk factor and an outcome of adiposity; this complex bidirectional association should be taken into account in future research and clinical practice.

## Key Points

**Question:**

Is cognitive function bidirectionally associated with adiposity among adolescents?

**Findings:**

In this cohort study that included 11 103 adolescents, executive function and episodic memory were bidirectionally associated with adiposity, and this association was statistically mediated through the morphology of the lateral prefrontal cortex.

**Meaning:**

These findings suggest that brain health should be taken into account as an outcome and risk factor associated with obesity in order to guide future research and clinical practice.

## Teaser

This cohort study assesses bidirectional associations between adiposity and cognitive function in youth in the United States and tests mediational pathways through the lateral prefrontal cortext.

## Introduction

Childhood obesity continues to be a significant public health concern worldwide. The World Health Organization reported that more than 18% of children and adolescents aged 5 to 19 years are either overweight or obese, representing approximately 340 million people worldwide.^1^ Many developed nations, including the US and Canada, have observed several-fold increases in obesity cases in the past few decades.^2,3,4^ Overweight children tend to remain overweight throughout their adulthood, and this excess adiposity substantially increases their risk of developing noncommunicable diseases, such as type 2 diabetes, heart diseases, and other chronic conditions at an early age.^2^

Obesity is negatively associated with several indicators of cognitive function in adult populations^5,6,7,8^; however, this relationship is less well studied in children and adolescents. A number of studies reported that excess adiposity in childhood is negatively associated with neurocognitive task performance.^9,10,11^ For example, obese children tend to perform worse on tasks of executive function, including those measuring facets of inhibitory control, mental flexibility, set-shifting, and verbal fluency.^9,10,11^ In contrast, mixed evidence has been found for impulsivity, planning, decision-making, reasoning, and sensitivity to reward.^9,10,11^ Furthermore, obesity is negatively associated with impaired attention, visuospatial performance, and motor skills among children and adolescents.^9,10,11^

Most studies on this topic have adopted a brain-as-outcome perspective, wherein it is assumed that adiposity leads to cognitive outcomes. However, within the health neuroscience perspective of obesity, the brain may serve as both risk factor and outcome in relation to adiposity.^12^ Prior theory and research findings have pointed to the possibility of such bidirectionality.^12,13,14^ For example, on average, overweight children have more limited inhibitory control compared with normal-weight children, which may potentiate excess caloric intake.^15^ Experimental research on young adult samples using transcranial magnetic brain stimulation (TMS) reveals that attenuation of lateral prefrontal function leads to increased food consumption, particularly when foods are hedonically appealing and environmental cues are permissive.^14,16^

Therefore, research findings to date suggest a possibility of a bidirectional association between adiposity and cognitive function with a prominent role of the prefrontal cortex (PFC) in mediating such associations. A recent cross-sectional analysis of the Adolescent Brain and Cognitive Development (ABCD) Study showed that the PFC morphologic features and adiposity indices are significantly associated with early adolescence^17^; however, the bidirectionality hypothesis and the mediating role of the PFC have not been explored. The current investigation aims to investigate the hypothesized bidirectional associations and potential mediation paths using the ABCD data set. We hypothesized that cognitive function and adiposity would be bidirectionally associated over time and that mediational mechanisms would differ depending on the directional pathway. Specifically, we anticipated that the association between baseline cognitive function and follow-up adiposity would be mediated by lateral PFC morphology and lifestyle behaviors, whereas the association between baseline adiposity and follow-up cognitive function would be mediated by physiological parameters, such as blood pressure. It was further hypothesized that bidirectionality would be most evident in relation to executive function but relatively consistent across adiposity metrics.

## Methods

### Data Source and Study Population

This cohort study made use of prospective cohort data from the ABCD Study,^18^ an ongoing longitudinal study of brain development in children in the US. The ABCD Study was launched in 2015 and recruited 11 878 children aged 9 to 10 years at 21 research sites in the US at inception.^19^ Data collection for the ABCD study is conducted on a half-yearly (brief telephone interview), annual (nonimaging), and biannual (imaging and bioassays) basis. This investigation used ABCD Data Release 4.0, which included wave 1 (baseline), wave 2 (1-year follow-up), and wave 3 (2-year follow-up) data sets. Detailed information about the study has been published elsewhere.^20,21^ All parents and children provided written informed consent and assent to take part in the ABCD Study. The present study received ethics approval from the Office of Research Ethics at the University of Waterloo. This study followed the Strengthening the Reporting of Observational Studies in Epidemiology (STROBE) reporting guideline.^22^

### Adiposity Indicators

#### Body Mass Index

In the ABCD protocol, participants’ height and weight were measured 3 times and averaged together.^23^ The body mass index (calculated as weight in kilograms divided by height in meters squared) z-score (zBMI) was calculated in accordance with the World Health Organization Child Growth Standards.^24^ BMI was measured at baseline (wave 1) and 2-year follow-up (wave 3) (eAppendix 1 in Supplement 1).

#### Waist Circumference (WC)

WC was measured around the abdomen at the level of the iliac crest using a tape measure. This measurement was taken once and provided in cm units.^23^ WC was measured during wave 1 and wave 3 data collection.

### Cognitive Function

#### NIH Toolbox Cognitive Battery

The National Institutes of Health (NIH) Toolbox is a comprehensive set of neurobehavioral tests that assesses motor, emotional, sensory, and cognitive function.^25^ The cognitive measures of NIH Toolbox, also known as NIH Toolbox Cognitive Battery, comprise 7 tasks that measure executive function, working memory, processing speed, attention, episodic memory, and language abilities (ie, vocabulary and phonologic processing).^21,25^ The advantage of the toolbox is that it is comprehensive, psychometrically sound, requires relatively short administration time, and is suitable for use in longitudinal studies. Furthermore, it can be used for a broad age range from 3 years onward and requires only 35 minutes to complete via tablet device. Two of the 7 tasks in the cognitive battery (ie, list sorting working memory and dimensional change card set) were not available at wave 3. Therefore, this study used the following 5 cognitive tasks for longitudinal assessments: the Flanker task (executive function), the pattern comparison processing speed test (speed of visual information processing), the picture sequence memory test (episodic memory), the picture vocabulary task (vocabulary) and the oral reading recognition task (phonologic processing). Higher scores on these tasks indicate better performance. All cognitive tasks were performed during wave 1 and wave 3 data collection (eAppendix 2 in Supplement 1).

#### Covariates and Mediators

The covariates selected for this study were age, sex, race and ethnicity, family income, parent education, area deprivation index, pubertal status, and sleep duration. Self-reported responses to the ABCD survey were used to derive information on race and ethnicity. Race and ethnicity were used as a covariate because some racial and ethnic backgrounds may have an association with socioeconomic deprivation, and accordingly, it can have an indirect relationship with brain development. Race and ethnicity were determined based on the self-reported responses to questionnaires (eg, Native American, Asian Indian, Black, Chinese, Filipino, Guamanian, Japanese, Korean, Native Hawaiian, other Asian, other Pacific Islander, Samoan, Vietnamese, White, and other race). We considered health behaviors (eg, diet, moderate-to-vigorous physical activity [MVPA]), blood pressure, and the morphology of the LPFC as putative mediators (Table 1 and eAppendix 3 in Supplement 1).

**Table 1.** Sample Characteristics^a^

Variables	No. (%)	Missing values
Total	11 103	
Wave 1 (baseline)		
Age, mean (SD), y	9.91 (0.6)	NA
Sex of child		NA
Male	5796 (52)	
Female	5307 (48)	
Child race		21
American Indian/Native American	69 (0.6)	
Asian Indian	55 (0.5)	
Black/African American	1828 (16)	
Chinese	89 (0.8)	
Filipino	47 (0.4)	
Guamanian	1 (<0.1)	
Japanese	13 (0.1)	
Korean	20 (0.2)	
Native Hawaiian	4 (<0.1)	
Other Asian	32 (0.3)	
Other Pacific Islander	14 (0.1)	
Other race^b^	477 (4.3)	
Samoan	4 (<0.1)	
Vietnamese	21 (0.2)	
White	8293 (75)	
Refuse	44 (0.4)	
Don’t know	71 (0.6)	
Hispanic ethnicity	2264 (21)	131
Puberty status, mean (SD)^c^	1.7 (0.8)	NA
Family income level, mean (SD)^c^	7.2 (2.4)	946
Primary parent education, mean (SD)^c^	16.6 (2.8)	12
Second parent/partner education, mean (SD)^c^	16.4 (3.0)	2284
Area deprivation index, mean (SD)	40.0 (26.9)	796
zBMI, mean (SD)	1.0 (2.4)	NA
Waist circumference, mean (SD), cm	26.5 (4.3)	8
Flanker task score, mean (SD)	46.0 (9.1)	9
Pattern matching score, mean (SD)	45.2 (14.4)	27
Picture sequence score, mean (SD)	49.5 (11.0)	17
Picture vocabulary score, mean (SD)	52.3 (11.0)	NA
Reading score, mean (SD)	49.3 (11.6)	17
Wave 2 (1-y follow-up)		
Diet^d^		
Whole grains	6436 (64)	1067
Green leafy vegetables	4671 (45)	832
Other vegetables	8690 (84)	749
Berries	6719 (66)	866
Beans	2723 (27)	837
Nuts	2384 (23)	874
Fast/fried food	6875 (66)	694
Pastries or sweets	6093 (59)	783
Wave 3 (2-y follow-up)		
Puberty status, mean (SD)^c^	2.5 (1.0)	1377
Blood pressure, mean (SD), mmHg		
Systolic	102.3 (10.7)	6894
Diastolic	60.4 (8.7)	6894
Physical activity, mean (SD), min	35.4 (32.1)	4691
Sleep duration, mean (SD), min	480.6 (80.0)	4860
zBMI, mean (SD)	1.9 (2.4)	4111
Waist circumference, mean (SD), cm	28.7 (4.8)	4138
Flanker task score, mean (SD)	46.7 (9.6)	3804
Pattern matching score, mean (SD)	54.5 (13.8)	3837
Picture sequence score, mean (SD)	52.9 (11.6)	1999
Picture vocabulary score, mean (SD)	49.7 (10.3)	2021
Reading score, mean (SD)	49.0 (10.7)	2058

Abbreviations: NA, not applicable; zBMI, body mass index (calculated as weight in kilograms divided by height in meters squared) z score.

^a^ The Adolescent Brain Cognitive Development Study participants were recruited from 21 study sites in the US (eTable 1 in Supplement 1).^19^

^b^ The category other race was chosen by those who felt they did not fit into major race subgroups. However, there was no additional information available regarding what falls under the other race category.

^c^ Higher scores on cognitive tasks indicate better performance. Pubertal status was derived from the Pubertal Developmental Scale, ranging from 1-4, where higher values indicate more pubertal maturity. Family income was recorded using a Likert-type scale, ranging from 1-10, where higher values indicate higher family income. Parent education was recorded using a Likert-type scale, ranging from 1-21, where higher values indicate a higher level of education.

^d^ No. (%) indicates the number of individuals and the percentage of people who confirmed consumption of respective items by selecting "Yes."

#### Brain Morphology

The lateral prefrontal cortex (LPFC) variables were derived from the ABCD structural magnetic resonance imaging (sMRI) module.^26^ The ABCD study consortium conducted and preprocessed all neuroimaging data. Morphologic features (eg, volume and thickness) of the LPFC and its subregions (eg, lateral orbitofrontal cortex [LOFC], middle frontal gyrus [MFG], and inferior frontal gyrus [IFG]) were estimated using Freesurfer version 5.3.0 (Freesurfer).^27^ Brain imaging was performed at wave 1 and wave 3 data collection. The methods of the brain imaging protocol are published elsewhere.^28^

### Statistical Analyses

Statistical analyses were performed using R version 4.1.0 (R Project for Statistical Computing).^29^ We used multivariate multivariable regression (MMR) and structural equation modeling (SEM) to assess the bidirectional associations between adiposity and cognitive function. Two-sided tests with 95% CIs and *P* < .05 were considered statistically significant. A total of 3 models were assessed in the MMR analyses where Model 1 was the unadjusted model, Model 2 was the basic demographics-adjusted model (ie, controlling for age, sex, parental education, and parental income), and Model 3 was the fully adjusted model (further control for race and ethnicity, ADI, pubertal status, and sleep duration). The brain-as-outcome path (adiposity to cognition) was analyzed using the wave 1 adiposity variable as the focal independent variable (ie, zBMI or WC) and all 5 wave 3 cognitive tasks together as dependent variables. A similar approach was also undertaken for the brain-as–risk factor path (cognition to adiposity), where each individual wave 1 cognitive variable was used as an independent variable, and 2 wave 3 adiposity indicators together were used as dependent variables.

**Figure 1.**
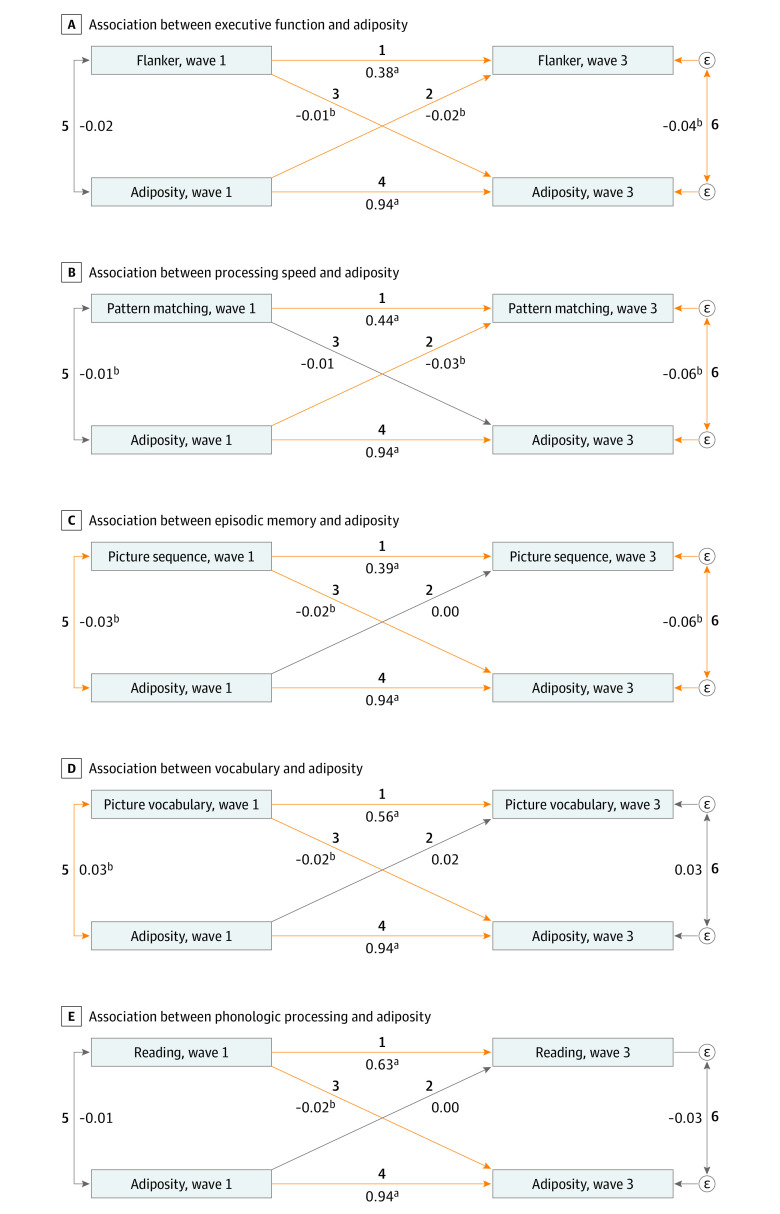
Cross-Lagged Model Estimates for Latent Adiposity and Cognitive Function Orange arrows indicate statistically significant path coefficients; gray arrows indicate nonsignificant path coefficients. All coefficients are standardized β weights. Covariates include age, sex, race and ethnicity, family income, parent education, area deprivation index, pubertal status, and sleep duration. In all panels, path 1 shows the association between baseline cognition and follow-up cognition; path 2 shows the association between baseline adiposity and follow-up cognition; path 3 shows the association between baseline cognition and follow-up adiposity; path 4 shows the association between baseline adiposity and follow-up adiposity; path 5 shows the covariance between baseline adiposity and baseline cognition; and path 6 shows the covariance between follow-up adiposity and follow-up cognition. Paths 2 and 3 (diagonal lines in the diagram) represent brain-as-outcome and brain-as–risk factor paths, respectively. Path 2 examined the strength of association between latent adiposity at baseline and cognition at follow-up, adjusting for the effects of baseline cognition and of the covariates. Similarly, path 3 examined the strength of association between cognition at baseline and latent adiposity at follow-up, adjusting for the effects of baseline adiposity and of the covariates. ^a^*P* < .05. ^b^*P* < .001.

Next, we constructed cross-lagged panel models with latent variable modeling (CLPM-L) for path analysis using the lavaan R package version 0.6-9 (R Project for Statistical Computing).^30^ Considering that the adiposity measures are strongly correlated (*r* = 0.78-0.87) and the cognitive measures mostly represent distinctive cognitive domains (*r* = 0.09-0.49), we used 2 adiposity indices (ie, zBMI and WC) to form a latent adiposity construct, whereas the cognitive variables were each used as a sole indicator of their respective cognitive domain in the SEM analyses (eFigure 1, eTable 2, and eTable3 in Supplement 1). The CLPMs are illustrated in Figure 1 and eFigure 1 in Supplement 1. In all CLPM-L models, including Figure 1, path 2 represents the prospective association between baseline adiposity and follow-up cognitive function and path 3 represents the prospective association between baseline cognitive function and follow-up adiposity. Paths 1 and 4 are associations between a given variable at baseline and the same variable at follow-up. Finally, paths 5 and 6 are cross-sectional associations between variables. Full information maximum likelihood estimation was used in the main set of models. Therefore, individuals who had at least baseline values for the variables of interest were included in the analyses irrespective of whether they had follow-up values or not. This approach is appropriate under the assumption that the data are missing at random.^31^

Finally, we analyzed potential mediation paths using the lavaan R package.^30^ Physical activity, diet, blood pressure, and brain morphology (ie, LPFC volume and thickness) were examined as mediators for both the brain-as-outcome path (adiposity to cognition) and the brain-as–risk factor path (cognition to adiposity). Parallel mediation models were used where a mediator constitutes multiple variables (eg, diet, blood pressure, and LPFC). Mediation analyses were adjusted for all Model 3 covariates described above.

## Results

A total of 11 103 individuals (mean [SD] age, 9.91 [0.6] years; 5307 females [48%]; 8293 White individuals [75%]; and 2264 Hispanic individuals [21%]) were included in the current study. Baseline and follow-up characteristics of study participants are presented in Table 1. Table 2 summarizes the findings of the MMR analyses. The analyses of path 2 (adiposity to cognition; brain-as-outcome) with baseline zBMI as an independent variable and 5 follow-up cognitive tasks together as the dependent variable suggested that higher zBMI was associated with worse performance on all cognitive tasks in the unadjusted models; however, this association was statistically significant in the fully adjusted model only for the picture sequence task (standardized estimate, β for model 1, −0.04; 95% CI, −0.06 to −0.02; β for model 2, −0.04; 95% CI, −0.07 to −0.02; β for model 3, −0.04; 95% CI, −0.07 to −0.01). This association was less consistent when baseline WC was used as a measure of adiposity, and only the association with follow-up picture vocabulary score emerged as statistically significant in the fully adjusted model (β for model 3, 0.03; 95% CI, 0.002 to 0.06), but in the opposite direction (ie, higher WC was associated with better scores on picture vocabulary task).

**Table 2.** Multivariate Multivariable Regression of the Analytic Sample for Path 2 (Adiposity to Cognition) and Path 3 (Cognition to Adiposity)^a^

Outcome	Model 1^b^		Model 2^c^		Model 3^d^	
	β (95% CI)	*P* value	β (95% CI)	*P* value	β (95% CI)	*P* value
Path 2						
zBMI to cognition						
Flanker	−0.04 (−0.06 to −0.02)	<.001	−0.04 (−0.06 to −0.01)	.004	0.03 (−0.06 to 0)	.07
Pattern matching	−0.03 (−0.05 to −0.01)	.01	−0.03 (−0.05 to 0)	.03	−0.03 (−0.06 to 0)	.06
Picture sequence	−0.04 (−0.06 to −0.02)	.001	−0.04 (−0.07 to −0.02)	<.001	−0.04 (−0.07 to −0.01)	.009
Picture vocabulary	−0.04 (−0.06 to −0.01)	.002	0.01 (−0.01 to 0.04)	.32	0.02 (−0.01 to 0.05)	.22
Reading	−0.05 (−0.08 to −0.03)	<.001	−0.02 (−0.04 to 0)	.11	−0.02 (−0.05 to 0.01)	.20
WC to cognition						
Flanker	−0.04 (−0.06 to −0.01)	.002	−0.02 (−0.05 to 0)	.06	−0.02 (−0.05 to 0.01)	.19
Pattern matching	−0.01 (−0.04 to 0.01)	.25	−0.01 (−0.04 to 0.01)	.30	−0.01 (−0.04 to 0.02)	.38
Picture sequence	−0.02 (−0.04 to 0)	.06	−0.03 (−0.06 to −0.01)	.01	−0.03 (−0.06 to 0)	.07
Picture vocabulary	−0.02 (−0.04 to 0.01)	.18	0.03 (0.01 to 0.06)	.008	0.03 (0 to 0.06)	.04
Reading	−0.02 (−0.05 to 0)	.05	0.01 (−0.02 to 0.03)	.64	0.00 (−0.03 to 0.03)	.90
**Path 3**						
Flanker to adiposity						
zBMI	−0.02 (−0.04 to 0.01)	.19	−0.02 (−0.04 to 0.00)	.07	−0.03 (−0.06 to −0.01)	.01
WC	−0.02 (−0.04 to 0)	.09	−0.03 (−0.05 to −0.01)	.02	−0.04 (−0.07 to −0.01)	.003
Pattern matching to adiposity						
zBMI	−0.02 (−0.04 to 0.00)	.09	−0.01 (−0.04 to 0.01)	.26	−0.01 (−0.03 to 0.02)	.60
WC	−0.01 (−0.03 to 0.01)	.48	−0.01 (−0.03 to 0.02)	.47	−0.01 (−0.03 to 0.02)	.56
Picture sequence to adiposity						
zBMI	−0.06 (−0.08 to −0.03)	<.001	−0.06 (−0.08 to −0.03)	<.001	−0.04 (−0.07 to −0.02)	.001
WC	−0.05 (−0.08 to −0.03)	<.001	−0.05 (−0.07 to −0.02)	<.001	−0.03 (−0.06 to 0)	.04
Picture vocabulary to adiposity						
zBMI	−0.04 (−0.06 to −0.02)	.001	−0.02 (−0.04 to 0.01)	.20	−0.02 (−0.04 to 0.01)	.26
WC	−0.02 (−0.05 to 0)	.07	0 (−0.03 to 0.02)	.87	0 (−0.03 to 0.02)	.73
Reading to adiposity						
zBMI	−0.06 (−0.08 to −0.04)	<.001	−0.03 (−0.05 to 0)	.04	−0.02 (−0.05 to 0)	.07
WC	−0.04 (−0.07 to −0.02)	<.001	−0.02 (−0.04 to 0.01)	.16	−0.02 (−0.05 to 0.01)	.14

Abbreviations: WC, waist circumference; zBMI, body mass index (calculated as weight in kilograms divided by height in meters squared) z score.

^a^ Higher scores on cognitive tasks indicate better cognitive status whereas higher scores on adiposity measures indicate poor adiposity status.

^b^ Model 1 is the unadjusted model.

^c^ Model 2 is the partially adjusted model controlled for age, sex, family income, and parent education.

^d^ Model 3 is the fully adjusted model controlled for age, sex, race and ethnicity, family income, parent education, area deprivation index, pubertal status, and sleep duration. All estimates are standardized coefficients.

The analyses of path 3 (cognition to adiposity; brain-as–risk factor perspective) with individual baseline cognitive tasks as independent variables and both follow-up adiposity measures as dependent variables suggested that better baseline scores on Flanker (zBMI: β for model 3, −0.03; 95% CI, −0.06 to −0.01; WC: β for model 3, −0.04; 95% CI, −0.07 to −0.01) and picture sequence tasks (zBMI: β for model 3, −0.04; 95% CI, −0.07 to −0.02; WC: β for model 3, −0.03; 95% CI, −0.06 to –0.002) were associated with lower follow-up adiposity in fully adjusted models. Picture vocabulary and reading tasks showed statistically significant associations in unadjusted and partially adjusted models, whereas pattern matching was not significantly associated with adiposity in any models (Table 2). Therefore, the bidirectional hypothesis was more well supported across models when picture sequence task was considered as an indicator of cognitive function and zBMI was considered as an indicator of adiposity.

**Figure 2.**
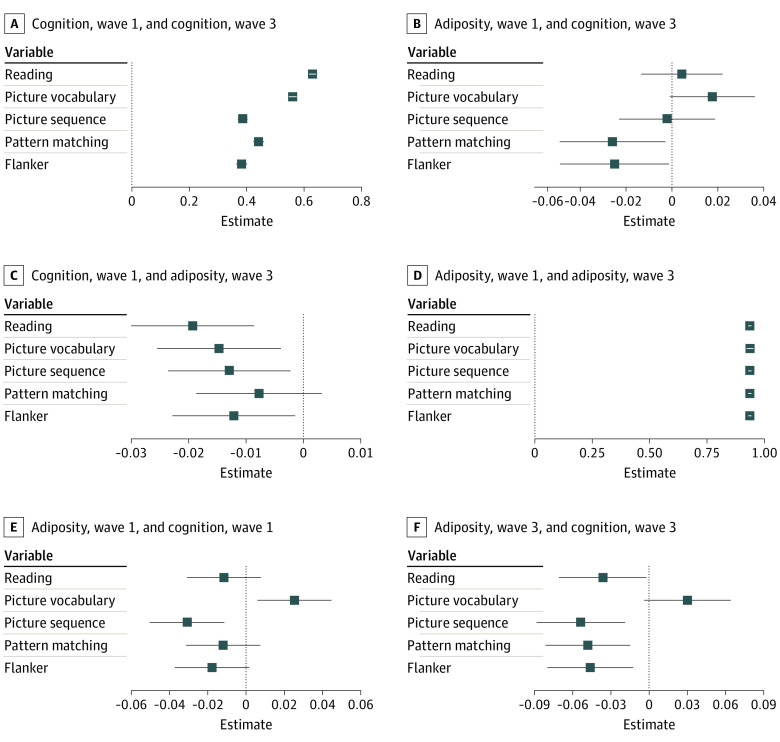
Bidirectional Associations Between Latent Adiposity and Cognitive Function Panels A (path 1) and D (path 4) indicate the associations between a given variable at baseline and the same variable at follow-up. Panel B (path 2) indicates the prospective association between baseline adiposity and follow-up cognitive function. Panel C (path 3) indicates the prospective association between baseline cognitive function and follow-up adiposity. Panel E (path 5) and F (path 6) indicate cross-sectional associations between variables. All estimates are standardized coefficients.

Table 3 summarizes the CLPM models testing prospective associations between each cognitive task as a sole indicator and latent adiposity constructed from zBMI and WC. The brain-as-outcome path (path 2; adiposity to cognition) revealed that lower baseline adiposity was associated with better follow-up Flanker interference (standardized estimate, β, −0.02; 95% CI, −0.05 to –0.001) and pattern comparison (*β,* −0.03; 95% CI, −0.05 to –0.003) task performance (Figure 1, Figure 2, Table 3, eFigure 2 in Supplement 1). On the other hand, the brain-as–risk factor path (path 3; cognition to adiposity) showed that better scores on Flanker (*β,* −0.01; 95% CI, −0.02 to –0.003), picture sequence (*β,* −0.02; 95% CI, −0.03 to –0.005), picture vocabulary (*β,* −0.02; 95% CI, −0.03 to –0.004) and oral reading (*β,* −0.02; 95% CI, −0.03 to −0.01) tasks were associated with significantly lower follow-up adiposity (Figure 1 and 2, Table 3; eFigure 2 in Supplement 1). Therefore, the bidirectional hypothesis was supported in relation to the Flanker task performance in the latent adiposity modeling. Sensitivity analyses were undertaken to examine the extent to which findings remained robust after adjusting for study site, and when underweight individuals (ie, individuals with a zBMI of less than 2 SD) were removed from the analysis. Results from these models revealed a similar overall pattern of findings (eFigure 3, eFigure 4, eTable 4, and eTable 5 in Supplement 1).

**Table 3.** Assessment of Bidirectional Relationship Between Latent Adiposity and Cognition^a^

Path label	Path description	Estimate (95% CI)	*P* value
**Flanker task**			
1	Cog_W1_ to Cog_W3_	0.38 (0.36 to 0.40)	<.001
2	Adi_W1_ to Cog_W3_	−0.02 (−0.05 to 0.00)	.04
3	Cog_W1_ to Adi_W3_	−0.01 (−0.02 to 0.00)	.02
4	Adi_W1_ to Adi_W3_	0.94 (0.93 to 0.95)	<.001
5	Adi_W1_ correlates Cog_W1_	−0.02 (−0.04 to 0.00)	.077
6	Adi_W3_ correlates Cog_W3_	−0.04 (−0.08 to −0.01)	.02
**Pattern comparison**			
1	Cog_W1_ to Cog_W3_	0.44 (0.42 to 0.46)	<.001
2	Adi_W1_ to Cog_W3_	−0.03 (−0.05 to 0.00)	.03
3	Cog_W1_ to Adi_W3_	−0.01 (−0.02 to 0.00)	.21
4	Adi_W1_ to Adi_W3_	0.94 (0.93 to 0.95)	<.001
5	Adi_W1_ correlates Cog_W1_	−0.01 (−0.03 to 0.01)	.23
6	Adi_W3_ correlates Cog_W3_	−0.06 (−0.09 to −0.02)	.002
**Picture sequence**			
1	Cog_W1_ to Cog_W3_	0.39 (0.37 to 0.40)	<.001
2	Adi_W1_ to Cog_W3_	0.00 (−0.02 to 0.02)	.83
3	Cog_W1_ to Adi_W3_	−0.02 (−0.03 to 0.00)	.006
4	Adi_W1_ to Adi_W3_	0.94 (0.93 to 0.95)	<.001
5	Adi_W1_ correlates Cog_W1_	−0.03 (−0.05 to −0.01)	.002
6	Adi_W3_ correlates Cog_W3_	−0.06 (−0.10 to −0.02)	.001
**Picture vocabulary**			
1	Cog_W1_ to Cog_W3_	0.56 (0.55 to 0.57)	<.001
2	Adi_W1_ to Cog_W3_	0.02 (0.00 to 0.04)	.06
3	Cog_W1_ to Adi_W3_	−0.02 (−0.03 to 0.00)	.008
4	Adi_W1_ to Adi_W3_	0.94 (0.93 to 0.95)	<.001
5	Adi_W1_ correlates Cog_W1_	0.03 (0.01 to 0.05)	.008
6	Adi_W3_ correlates Cog_W3_	0.03 (−0.01 to 0.07)	.10
**Oral reading recognition**			
1	Cog_W1_ to Cog_W3_	0.63 (0.62 to 0.64)	<.001
2	Adi_W1_ to Cog_W3_	0.00 (−0.01 to 0.02)	.60
3	Cog_W1_ to Adi_W3_	−0.02 (−0.03 to −0.01)	.001
4	Adi_W1_ to Adi_W3_	0.94 (0.93 to 0.95)	<.001
5	Adi_W1_ correlates Cog_W1_	−0.01 (−0.03 to 0.01)	.27
6	Adi_W3_ correlates Cog_W3_	−0.03 (−0.07 to 0.01)	.10

Abbreviations: Adi_W1_, latent adiposity variable at baseline (wave 1); Adi_W3_, latent adiposity variable at follow-up (wave 3); Cog_W1_, cognition at baseline (wave 1); Cog_W3_, cognition at follow-up (wave 3).

^a^ Higher scores on cognitive tasks indicate better cognitive status whereas higher scores on adiposity measure indicate worse adiposity status. The analyses were adjusted for age, sex, race and ethnicity, family income, parent education, area deprivation index, pubertal status, and sleep duration. All estimates are standardized coefficients.

The findings of the mediation analyses are summarized in eTable 6 and eTable 7 in Supplement 1. Path 2 mediation analyses suggested that the association between baseline adiposity and follow-up reading performance was statistically mediated through systolic BP (zBMI: *b,* −0.0408; 95% CI, −0.0768 to −0.0048; WC: *b,* −0.0256; 95% CI, −0.0470 to −0.0042) and MFG volume (zBMI: *b,* −0.0073; 95% CI, −0.0143 to −0.0003; WC: *b,* −0.0051; 95% CI, −0.0099 to −0.0004). A statistically significant mediation path was also observed through MFG thickness (zBMI: *b,* −0.0183; 95% CI, −0.0331 to −0.0035; WC: *b,* −0.0061; 95% CI, −0.0113 to −0.0009) and IFG thickness (zBMI: *b,* 0.0142; 95% CI, 0.0012 to 0.0272; WC: *b,* 0.0065; 95% CI, 0.0007 to 0.0123) for baseline adiposity and follow-up picture vocabulary task performance. MFG thickness also emerged as a statistically significant mediator for the association between baseline adiposity and follow-up pattern recognition task performance (zBMI: *b, *0.0373; 95% CI, 0.0143 to 0.0602; WC: *b,* 0.0117; 95% CI, 0.0036 to 0.0199). Physical activity emerged as a significant mediator for the association between baseline adiposity and follow-up pattern recognition (zBMI: *b,* −0.0387; 95% CI, −0.0687 to −0.0086; WC: *b,* −0.0192; 95% CI, −0.0334 to −0.005), picture vocabulary (zBMI: *b,* −0.0489; 95% CI, −0.0692 to −0.0286; WC: *b,* −0.0224; 95% CI, −0.0321 to −0.0127) and oral reading (zBMI: *b,* −0.0214; 95% CI, −0.0406 to −0.0022; WC: *b,* −0.0108; 95% CI, −0.0199 to −0.0017) task performance.

On the other hand, path 3 analyses showed that the association between baseline cognitive function and follow-up adiposity was statistically mediated through physical activity for the picture vocabulary task (zBMI: *b,* −0.0011; 95% CI, −0.0015 to −0.0007; WC: *b,* −0.0020; 95% CI, −0.002 to −0.0012) and the oral reading task (zBMI: *b,* −0.0007; 95% CI, −0.0010 to −0.0003; WC: *b,* −0.0013; 95% CI, −0.0020 to −0.0007). In addition, LPFC thickness was a significant mediator for the association between baseline pattern recognition and follow-up WC (*b, *0.0004; 95% CI, 0.0001 to 0.0006).

## Discussion

This cohort study aimed to test the possibility of a bidirectional association between adiposity and cognitive function in adolescents using a large population-based sample. Our fully adjusted regression analyses suggested that higher baseline adiposity was associated with lower performance on an episodic memory task (picture sequence) at follow-up. Latent adiposity modeling showed statistically significant inverse associations between baseline adiposity and follow-up executive function (Flanker task) and visual processing speed (pattern matching), thereby supporting the brain-as-outcome perspective in relation to adiposity and cognitive function. When considering the brain-as–risk factor perspective, better baseline executive function and episodic memory were found to be associated with lower adiposity at follow-up in regression models. Latent adiposity modeling showed statistically significant inverse associations between adiposity and all cognitive variables except processing speed. Overall, when considering latent adiposity modeling, the brain-as–risk factor perspective appears to be more well-supported in the adolescent age group compared with the brain-as-outcome perspective (although the latter received some limited support as well). However, our latent variable findings confirmed bidirectionality with respect to executive function, as hypothesized. Finally, mediation analyses suggested significant indirect paths through blood pressure, physical activity, and LPFC volume and thickness.

The findings of this investigation are largely consistent with previous research conducted on adults and youths. Previous studies that assessed the brain-as-outcome path unidirectionally reported that excess adiposity is negatively associated with several domains of cognitive function in adults (eg, short-term memory, psychomotor function, selective attention, decision-making, planning, and problem-solving), with a more pronounced association observed in the domain of executive function.^5,32^ Similar to adult populations, excess adiposity was also reported to be associated with cognitive performance decrements in children and adolescents.^9,10^ Consistent with our findings, previous studies reported adiposity among youth is associated with lower processing speed,^33^ weaker executive control,^34^ and suboptimal episodic memory^35^.

Although incompletely explored, the results of previous longitudinal investigations support the brain-as–risk factor perspective. For example, it has been reported that children and adolescents with relatively weaker executive control, poorer planning, and more impulsivity at baseline were more likely to have high BMI at follow-up.^36,37,38,39^ The hypothesized bidirectional association between adiposity and cognitive function has also been reported in previous studies using adult samples.^7^ A recent meta-analysis of longitudinal studies involving children and adolescent samples showed that the existing literature supports a bidirectional relationship between adiposity and executive function.^11^ However, it should be noted that meta-analyses included studies that assessed adiposity to executive function or executive function to adiposity associations primarily with a unidirectional assumption, mostly in different studies. Therefore, the bidirectional associations concluded in the meta-analysis were observed in different samples and over different time windows. On the other hand, our investigation showed that the hypothesized bidirectional associations can be observed in the same population and timeframe in parallel, making the current findings an important and novel contribution to the existing literature.

With respect to mediation analyses, we observed that MFG volume and thickness emerged as significant mediators, primarily for the brain-as-outcome path. Previous studies also reported a link between brain morphology and cognitive development in several domains. For example, using the same data set, Ronan et al^40^ found that higher BMI was associated with reduced PFC thickness in adolescents and such cortical thinness partially accounted for subnormal executive control. Likewise, Hall et al^17^ reported that cortical thickness of PFC was significantly associated with multiple indices of adiposity (eg, zBMI and WC). Additionally, as reported in previous studies, excess adiposity can also adversely affect other brain structures, such as the hippocampus^41^ and the amygdala.^42^

With respect to other hypothesized mediators, not all associations were in the expected direction. For instance, although a significant indirect mediation path involving MVPA was found, the direction of the association suggested that higher vocabulary and reading scores were associated with lower MVPA. This negative association might reflect time competition between academic pursuits and physical activity, such that more hours spent studying (a sedentary activity) might produce higher scores on tests of reading and vocabulary, and produce a negative correlation between cognitive task performance and MVPA.^43^ The null mediation paths from wave 1 cognition to wave 3 adiposity should be considered tentative, given the assumed gradual, cumulative nature of the association between baseline cognition and future body composition. A longer time window would be ideally suited to test this mediational pathway adequately.

This study has several strengths. First, the large sample size ensured high statistical power to detect subtle associations that would be expected to be barely discernable over a relatively brief developmental timeframe (ie, <5 years). Second, given that the ABCD cohort was amassed from 21 geographically dispersed research sites across the host country, it could be considered to be broadly representative of the US. Third, the direct measurement of anthropometrics reduces the possibility of self-report biases.^44^ Fourth, the current analyses were adjusted for a number of sociodemographic variables that could potentially confound the hypothesized bidirectional association. Fifth, we were able to examine the mediation paths using brain morphologic data assessed by structural brain imaging, a unique facet of this investigation.

### Limitations

This study had limitations. First, survey weights were not constructed and implemented for this study, so the findings may not be precisely representative of the larger population of the US. Second, although the ABCD data set is rich in cognitive assessments, there are only 2 measures available for adiposity indicators over the wave 1 to wave 3 period. Therefore, it was not possible to test the bidirectionality hypothesis using other desirable measurements, such as dual-energy x-ray absorptiometry and waist-to-hip ratio. Third, the CLPM-L approach undertaken to test bidirectionality depends on several assumptions, which are often violated or cannot be entirely met.^45^ Finally, at the time of this investigation, adiposity and cognitive variables were measured only at 2 waves (wave 1 or baseline and wave 3), leading to insufficient data to conduct more preferable modeling strategies, such as latent growth curve modeling (eAppendix 5 in Supplement 1). Furthermore, the brief interval between wave 1 and wave 3 was barely sufficient for the accumulation of changes in cognition or adiposity in this age group. More follow-up data with an extended period of follow-up are required to more conclusively evaluate the true magnitude of the hypothesized bidirectional associations.

Despite the limitations mentioned above, this study provides some valuable insights on bidirectional associations that could be expected in the adolescent population. Further studies are needed to replicate the findings, particularly in children, adolescents, and youth populations. Future studies should also aim to include other adiposity measures and additional cognitive variables to detect to what extent bidirectional associations exist beyond executive function and episodic memory.

## Conclusions

In this cohort study, we tested the possibility of bidirectional associations between cognitive function and adiposity using a large population-based sample of adolescents. Although the findings varied slightly based on the modeling approach, we observed bidirectional associations of adiposity with episodic memory and executive function. Findings of mediation analyses were less consistent; however, significant mediation paths were observed for blood pressure, physical activity, and MFG volume and thickness, depending on the direction of the association. The finding that the brain is both a product of and an influence on adiposity in adolescence is consistent with modern perspectives on obesity and the brain in early life.^46^ The implications for the management and prevention of obesity suggest that weight management through exercise and eating are important, as well as activities that preserve and optimize brain health for its own sake.

## Notes

Supplement 1. **eAppendix 1.** Body Mass Index **eAppendix 2.** Description of Cognitive Tasks **eAppendix 3.** Covariates and Mediators **eAppendix 4.** Description of Limitations Because of the Use of Default Atlas for MRI **eAppendix 5.** Description of Limitations in Cross-Lagged Panel Model and of Alternative Models **eFigure 1. **Conceptual Diagram of the Bidirectional Associations Between Adiposity and Cognition **eFigure 2.** Bidirectional Associations Between Latent Adiposity and Cognitive Function (Main Analysis With Point Estimates) **eFigure 3.** Bidirectional Associations Between Latent Adiposity and Cognitive Function (Sensitivity Analysis 1) **eFigure 4.** Bidirectional Associations Between Latent Adiposity and Cognitive Function (Sensitivity Analysis 2) **eTable 1.** Description Of Study Sites **eTable 2.** Pearson Product-Moment Correlations Among Cognitive Variables **eTable 3.** Pearson Product-Moment Correlations Among Adiposity Variables **eTable 4.** Sensitivity Analysis by Removing Winsorization and Excluding Underweight Individuals **eTable 5.** Sensitivity Analysis by Including Study Sites and Excluding Pubertal Status and Sleep Duration **eTable 6.** Mediation Analysis for Path 2 **eTable 7.** Mediation Analysis for Path 3
eReferences.

Supplement 2.
Data Sharing Statement
