# Errors in Units

**DOI:** 10.1001/jamanetworkopen.2023.46385

**Published:** 2023-11-21

In the Original Investigation titled “Bidirectional Associations Between Adiposity and Cognitive Function and Mediation by Brain Morphology in the ABCD Study,”^1^ published February 16, 2023, there are errors in the units of waist circumference in Table 1 and in the Methods section. There units were written as centimeters but should be inches. This article has been corrected.^1^
