# Supplementary material for: Bidirectional Associations Between Adiposity and Cognitive Function and Mediation by Brain Morphology in the ABCD Study
Source: JAMA Netw Open. 2023 Feb 16;6(2):e2255631. doi: 10.1001/jamanetworkopen.2022.55631 (PMC9936350; doi:10.1001/jamanetworkopen.2022.55631)
Supplement: Supplement 1. — eAppendix 1. Body Mass Index eAppendix 2. Description of Cognitive Tasks eAppendix 3. Covariates and Mediators eAppendix 4. Description of Limitations Because of the Use of Default Atlas for MRI eAppendix 5. Description of Limitations in Cross-Lagged Panel Model and of Alternative Models eFigure 1. Conceptual Diagram of the Bidirectional Associations Between Adiposity and Cognition eFigure 2. Bidirectional Associations Between Latent Adiposity and Cognitive Function (Main Analysis With Point Estimates) eFigure 3. Bidirectional Associations Between Latent Adiposity and Cognitive Function (Sensitivity Analysis 1) eFigure 4. Bidirectional Associations Between Latent Adiposity and Cognitive Function (Sensitivity Analysis 2) eTable 1. Description Of Study Sites eTable 2. Pearson Product-Moment Correlations Among Cognitive Variables eTable 3. Pearson Product-Moment Correlations Among Adiposity Variables eTable 4. Sensitivity Analysis by Removing Winsorization and Excluding Underweight Individuals eTable 5. Sensitivity Analysis by Including Study Sites and Excluding Pubertal Status and Sleep Duration eTable 6. Mediation Analysis for Path 2 eTable 7. Mediation Analysis for Path 3 eReferences. [file jamanetwopen-e2255631-s001.pdf]

Supplemental Online Content

Sakib MN, Best JR, Hall PA. Bidirectional associations between adiposity and cognitive function and mediation by brain morphology in the ABCD study. *JAMA Netw Open*. 2023;6(2):e2255631. doi:10.1001/jamanetworkopen.2022.55631

eAppendix 1. Body Mass Index

eAppendix 2. Description of Cognitive Tasks

eAppendix 3. Covariates and Mediators

eAppendix 4. Description of Limitations Because of the Use of Default Atlas for MRI

eAppendix 5. Description of Limitations in Cross-Lagged Panel Model and of Alternative Models

eFigure 1. Conceptual Diagram of the Bidirectional Associations Between Adiposity and Cognition

eFigure 2. Bidirectional Associations Between Latent Adiposity and Cognitive Function (Main Analysis With Point Estimates)

eFigure 3. Bidirectional Associations Between Latent Adiposity and Cognitive Function (Sensitivity Analysis 1)

eFigure 4. Bidirectional Associations Between Latent Adiposity and Cognitive Function (Sensitivity Analysis 2)

eTable 1. Description of Study Sites

eTable 2. Pearson Product-Moment Correlations Among Cognitive Variables

eTable 3. Pearson Product-Moment Correlations Among Adiposity Variables

eTable 4. Sensitivity Analysis by Removing Winsorization and Excluding Underweight Individuals

eTable 5. Sensitivity Analysis by Including Study Sites and Excluding Pubertal Status and Sleep Duration

eTable 6. Mediation Analysis for the Path 2

eTable 7. Mediation Analysis for Path 3

eReferences.

This supplemental material has been provided by the authors to give readers additional information about their work.

© 2023 Sakib MN et al. *JAMA Network Open.*

eAppendix 1. Body mass index

BMI was calculated from average height and weight and converted to z-scores (zBMI) in accordance with the World Health Organization Child Growth Standards (1). The conversion was done in R using ‘zscorer’ package (2). To deal with the extreme values, winsorization was applied such that values below the 1st and above the 95th percentile were set to those percentile values, respectively. Winsorization was applied asymmetrically because there were more extreme values at the positive end than the negative end. At wave 1, the 1st percentile corresponded to a BMI z-score of -2.99 and the 95th percentile was 5.83; at wave 3, these values were -2.35 and 7.33, respectively.

© 2023 Sakib MN et al. *JAMA Network Open.*

eAppendix 2. Description of cognitive tasks

Flanker Task

The Flanker paradigm primarily measures executive function, particularly inhibitory control and attention (3, 4). It assesses participants' ability to suppress tempting, but irrelevant responses to a given context. In the NIH Toolbox version of the task, participants are shown a row of five arrows on each trial (4). The outer 4 arrows are called distractors or "flankers", all of which are pointed in the same direction (right or left). Participants need to identify the direction of the middle arrow (the target), which either pointed towards the same direction as the flankers (congruent trial) or the opposite direction of the flankers (incongruent trial). Participants register their responses by pressing one of the two arrows displayed on the screen. The task is implemented using fish icons with arrows as stimuli in the case of younger children (3-6 years) (5). Given the older minimum age of 9-10 years in ABCD, the arrow version of the task was used. The variable of interest is flanker interference calculated by taking the difference between reaction time of correct incongruent and congruent trials. Higher flanker interference indicates poor ability to suppress the distracting stimuli, and accordingly, it suggests weaker control of executive function, particularly the inhibitory control domain. For the current analyses, Flanker scores were reversed coded such that higher scores indicate better executive control. The NIH toolbox version of the Flanker task showed excellent test-retest reliability (Intraclass Correlation Coefficients [ICC] = 0.95) and acceptable convergent (r = -0.48) and discriminant validity (r = 0.15) comparing the gold standard (6).

Pattern Comparison Processing Speed Test

This task is designed to measure the speed of visual processing (7-9). In this task, participants are shown two images side-by-side and asked to determine whether the images are identical or not. Nonidentical patterns are varied by one of three dimensions: color, adding/taking something away, or one versus many (9). Participants register their response by pressing a "yes" or "no" button on the screen. The test score calculated as the total number of correct answers in 90 seconds. In terms of psychometric properties, high test-retest reliability (ICC = 0.82) and acceptable convergent and discriminant validity were shown in previous studies (6).

Picture Sequence Memory Test

This task assesses episodic memory and involves acquiring, storing and recalling new information (10, 11). It can be administered on participants aged between 3 and up. In this task, participants are presented with a sequence of pictures that depicts activities or events; an audio clip is played simultaneously to describe the contents briefly. Participants are asked to reproduce the sequence in the order it was shown. The sequence length is adjusted based on the age of the participants and can be varied between 6-18 images. One point is awarded for correctly placing each adjacent pair of the sequence. Therefore, the final score indicates the total number of adjacent pairs remembered accurately by each participant. This task also showed good test-retest reliability (ICC = 0.78) and acceptable convergent and discriminant validity (6).

© 2023 Sakib MN et al. *JAMA Network Open.*

Picture Vocabulary Task

This test is a modified version of the Peabody Picture Vocabulary Test (PPTV) and assesses receptive vocabulary and language comprehension of an individual (12, 13). In this test, children hear an audio clip of a word while observing four photographic images (of objects, actions and/or depictions of concepts) in a square on the screen. Children are instructed to press the image that closely matches the meaning of the word. Items are scored as correct or incorrect. This test implements computerized adaptive testing (CAT) in order to ensure appropriate item difficulty. The test-retest reliability (ICC = 0.94), convergent and discriminant validity reported for this task were acceptable (6).

Oral Reading Recognition Task

This task measures exposure to language materials and cognitive skills involved in reading (12, 13). In the task, participants are presented with a series of words on the screen and instructed to pronounce them as accurately as possible. This task uses CAT to ensure appropriate item difficulty. Good test-retest reliability (ICC = 0.99) and acceptable convergent and discriminant validity were also reported for this task in previous investigations (6).

© 2023 Sakib MN et al. *JAMA Network Open.*

eAppendix 3. Covariates and mediators

Demographic factors

Age. Participants' age in months at the time of the interview was recorded and rounded to the nearest chronological month (14).

Sex. Participant's sex at birth was recorded and coded as M = Male, F = Female, O = Other, NR = Not reported (14).

Race. The ABCD study participants were from various ethnic and racial backgrounds (14). See Table 1 for the list of the ethnic background of the participants.

Child Hispanic ethnicity. Parents were asked "Do you consider the child Hispanic/Latino/Latina?" (14). This variable was coded as 1 = Yes and 2 = No.

Family income. Family income was defined as the combined income of all adults in home. Participants were asked "What is your total combined family income for the past 12 months?" with the options of the following categories provided: 1= Less than $5,000; 2=$5,000 through $11,999; 3=$12,000 through $15,999; 4=$16,000 through $24,999; 5=$25,000 through $34,999; 6=$35,000 through $49,999; 7=$50,000 through $74,999; 8= $75,000 through $99,999; 9=$100,000 through $199,999; 10=$200,000 and greater (14).

Primary parent education. The parent was asked, "What is the highest grade or level of school you have completed or the highest degree you have received?" The response to this question constitutes 21 categories with 1 being the "Never attended/Kindergarten only" to 21 being the "Doctoral degree", and the rest of the intermediate categories represented other education levels in between in the ascending order of the hierarchy (e.g., grade 1-12, high school graduate, GED or equivalent Diploma, Some college, Associate degree: Occupational, Associate degree: Academic Program, Bachelor's degree, Master's degree, and Professional School degree) (14).

Area deprivation index (ADI). The ADI is a multidimensional tool to assess socioeconomic disadvantage of neighborhoods constructed using income, education, employment, and housing status of the regions (15). Such index was previously found to have an association with health outcomes and obesity (16). The ADI score was provided in the dataset as a percentile, with higher values representing more deprivation (14). This score was used as a continuous variable in the covariate-adjusted models.

Health Status and Behavioral Factors

Pubertal status. Parents and children were asked several general and sex-specific questions (e.g., body hair, voice change, skin change, facial hair, menstrual history, etc.) to understand the pubertal status of the participants. The Pubertal Developmental Scale was created for boys and girls using the responses to those questions with the following categories: 1-prepuberty, 2-early puberty, 3-mid puberty, 4-late puberty, and 5-post puberty (14).

Moderate-to-vigorous physical activity (MVPA). The data on physical activity were derived from the weekly physical activity summaries of a wrist-worn tri-axial accelerometer using a commercial Fitbit device (14). Average minutes spent in moderate activity (3-5.9 metabolic equivalent of task [METS]) and vigorous activity (6+ METS) during day were

© 2023 Sakib MN et al. *JAMA Network Open.*

provided as two different measures. A sum of these measures was taken to create the MVPA variable.

Sleep duration. The sleep duration was also taken from the wrist-worn accelerometer device. Sleep periods were defined using the average of all minutes classified as being any kind of sleep (i.e., light + deep + REM) for all included days, based on (low) movement counts (14).

Diet. Dietary behaviors were assessed in the “ABCD Child Nutrition Assessment” module (14). Intake of whole grains, green leafy vegetables, other vegetables, berries, beans and nuts were coded as “healthy” food choices. To query the intake of healthy foods, parents of the participants were asked: “In a typical week, does your child eat (i) Whole grains 3 or more times per day, (ii) Green leafy vegetables 6 or more times per week, (iii) Other vegetables 1 or more time per day, (iv) Berries 2 or more times per week”, (v) Beans 4 or more times per week, and (vi) Nuts 5 or more times per week? Responses for each of these probes were coded as 1 = Yes or 0 = No.

Intake of fast food and pastries were coded as “unhealthy” food choices. To query intake patterns of unhealthy foods, parents were asked: “In a typical week, does your child eat (i) fast food or fried food less than 1 time per week, (ii) Pastries or sweets less than 5 times per week? These variables were also coded as 1 = Yes and 0 = No (14).

Blood pressure (BP). Systolic and diastolic BP was measured three times at wave 3 data collection (14). The average of systolic BP and the average of diastolic BP were provided separately in the dataset as continuous variables.

© 2023 Sakib MN et al. *JAMA Network Open.*

eAppendix 4. Description of limitations because of the use of default atlas for MRI

Morphological features of the cortex were estimated using Freesurfer v5.3.0 (17). Freesurfer utilizes an automated, atlas-based, volumetric segmentation procedure for cortical surface reconstruction and subcortical segmentation. Images obtained from reconstruction were visually inspected, and only the images of sufficient quality were included in the study. Finally, morphological features were processed for the Deskian-Killiany atlas as part of the standard FreeSurfer pipeline (18). The use of the default atlas could have some influence on the mediation analyses; therefore, a stronger or weaker mediational effect could be observed using other atlases.

© 2023 Sakib MN et al. *JAMA Network Open.*

eAppendix 5. Description of limitations in cross-lagged panel model and of alternative models

With only two time points of data available during which cognition and adiposity were measured, the only type of dynamic, bidirectional model that can be identified is the cross-lagged panel model (CLPM) employed in the current study. With additional waves of data collection, more sophisticated models can be identified, including the random intercept-CLPM (19) and the dual change score model (20). In short, these models are capable of distinguishing between- from within-person effects, as well as modeling group-level change over time in the constructs of interest, both of which are not possible when applying the CLPM to two waves of data. For a more comprehensive discussion and comparison of these models, see Mund and Nestler (2019) (21). All that said, the CLPM is an important tool for the behavioural researcher that allows one to address questions such as: "Does a child with a high level of adiposity (relative to others) experience a subsequent lower development in cognition?" Furthermore, more sophisticated models often suffer from poor model convergence and/or only examine prospective within-person effects (22).

© 2023 Sakib MN et al. *JAMA Network Open.*

eFigure 1. Conceptual diagram of the bidirectional associations between adiposity and cognition

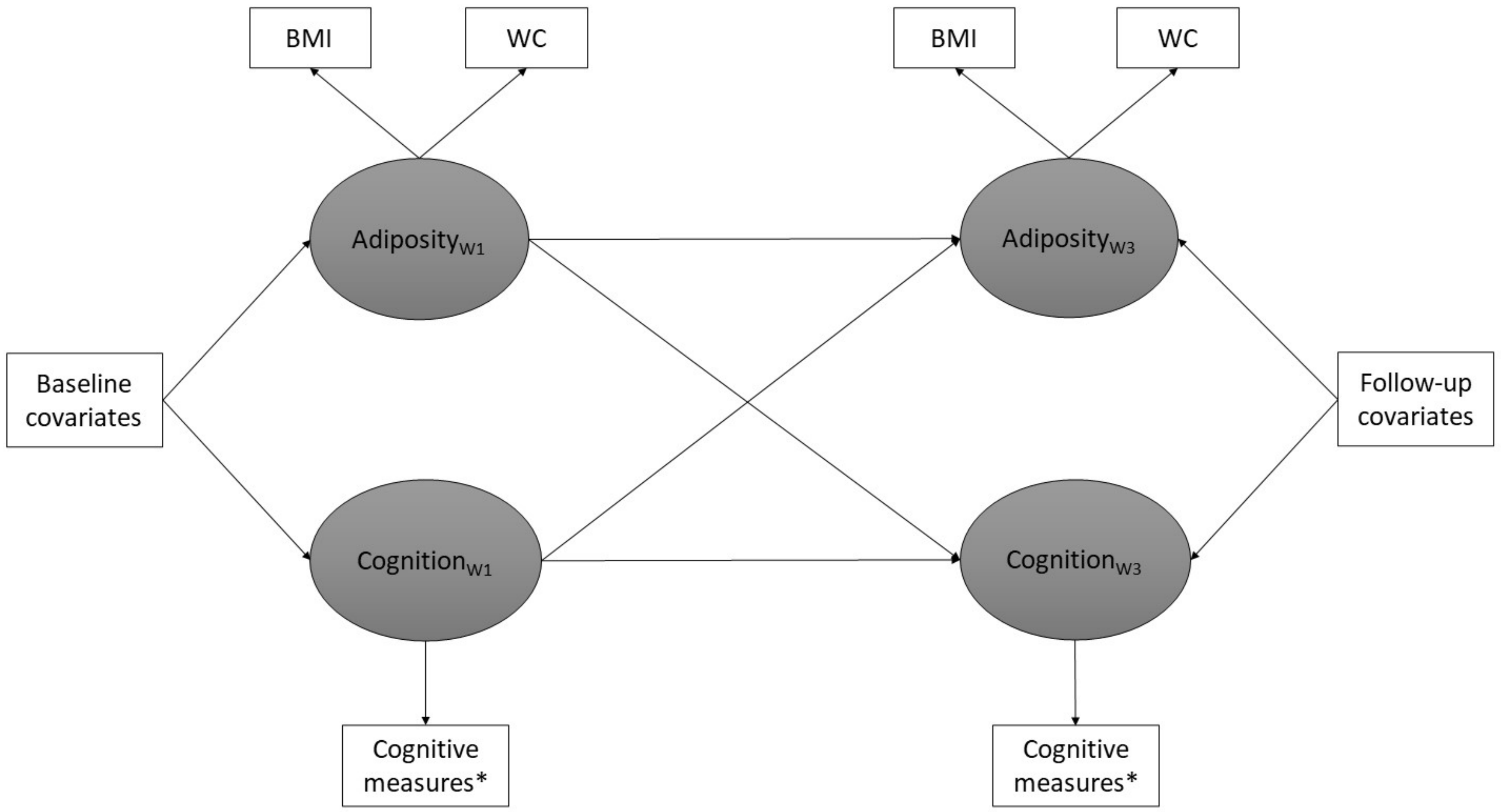

Note: WC = Waist Circumference, BMI = Body Mass Index, W1 = Wave 1 or baseline measures, W3 = Wave 3 or follow-up measures. *A separate cognitive measure was used in each model and included the following NIH Toolbox tasks: Flanker task, pattern matching, picture sequence, picture vocabulary and reading tasks. The covariates considered for the bidirectional associations were age, sex, ethnicity, family income, parent education, area deprivation index, pubertal status and sleep duration.

© 2023 Sakib MN et al. *JAMA Network Open.*

eFigure 2. Bidirectional associations between latent adiposity and cognitive function (main analysis with point estimates)

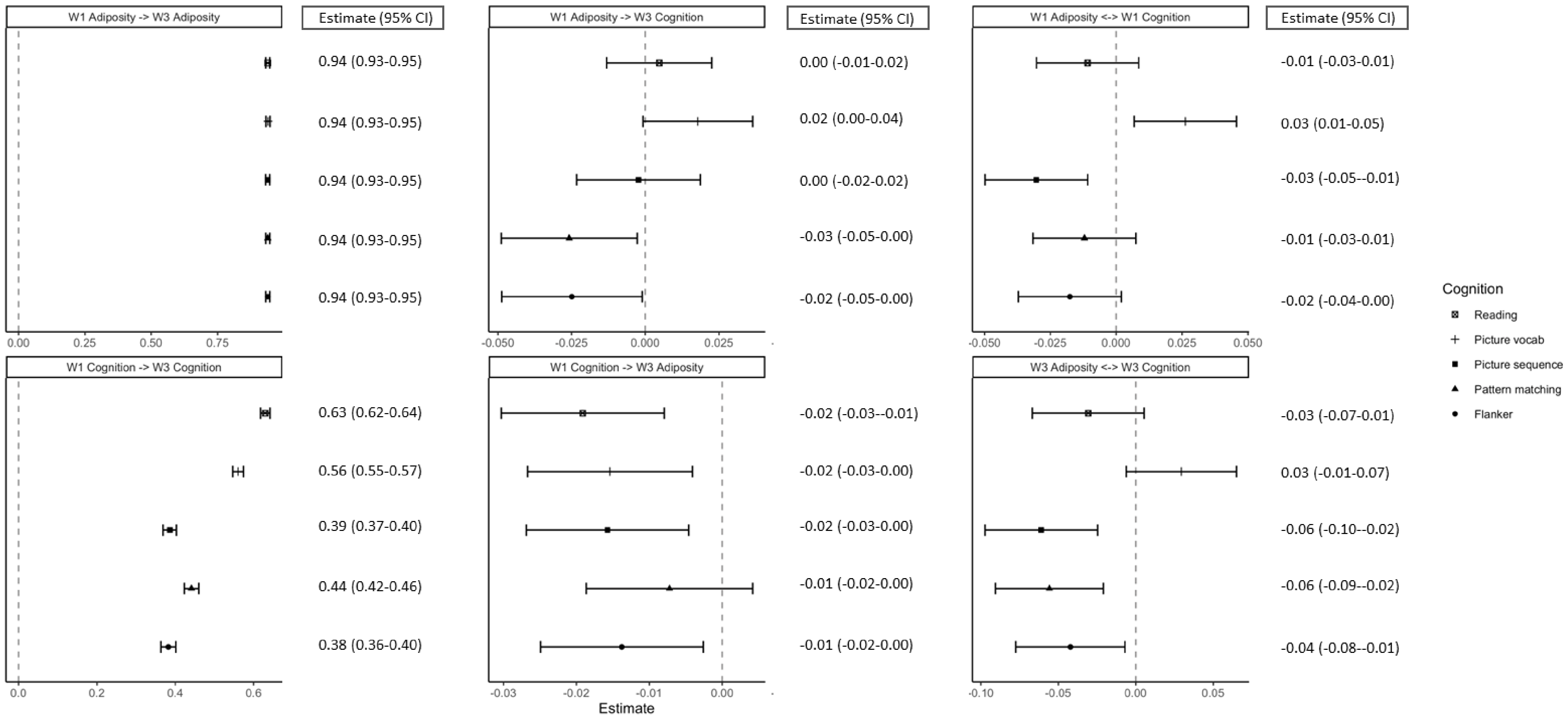

Note: W1 = Wave 1 or baseline measures; W3 = Wave 3 or 2-year follow-up measures; Adiposity = Latent adiposity variable.

© 2023 Sakib MN et al. *JAMA Network Open.*

eFigure 3. Bidirectional associations between latent adiposity and cognitive function (sensitivity analysis 1)

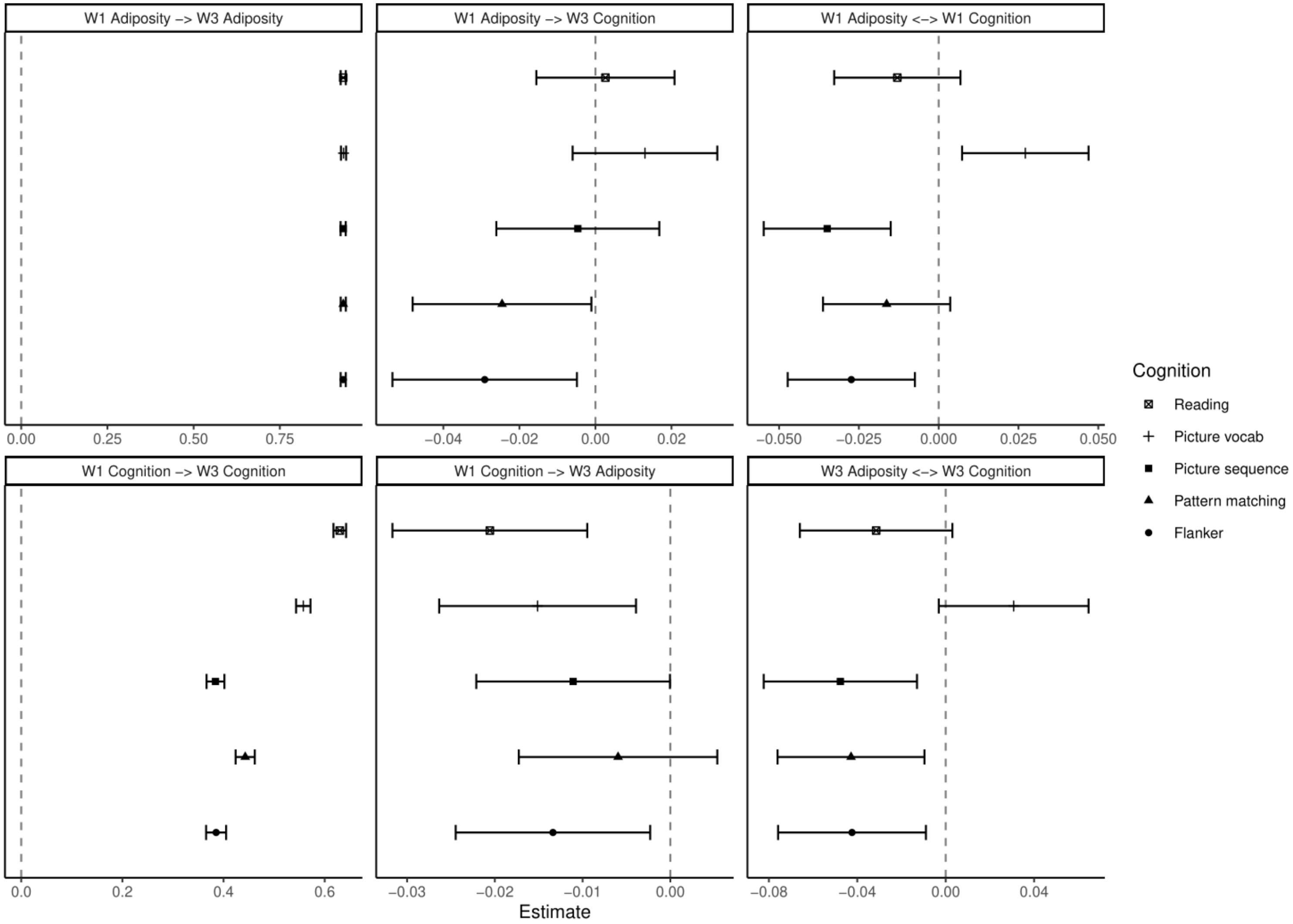

Note: Sensitivity analysis was conducted by removing winsorization and excluding underweight individuals from the analysis. W1 = Wave 1 or baseline measures; W3 = Wave 3 or 2-year follow-up measures; Adiposity = Latent adiposity variable.

© 2023 Sakib MN et al. *JAMA Network Open.*

eFigure 4. Bidirectional associations between latent adiposity and cognitive function (sensitivity analysis 2)

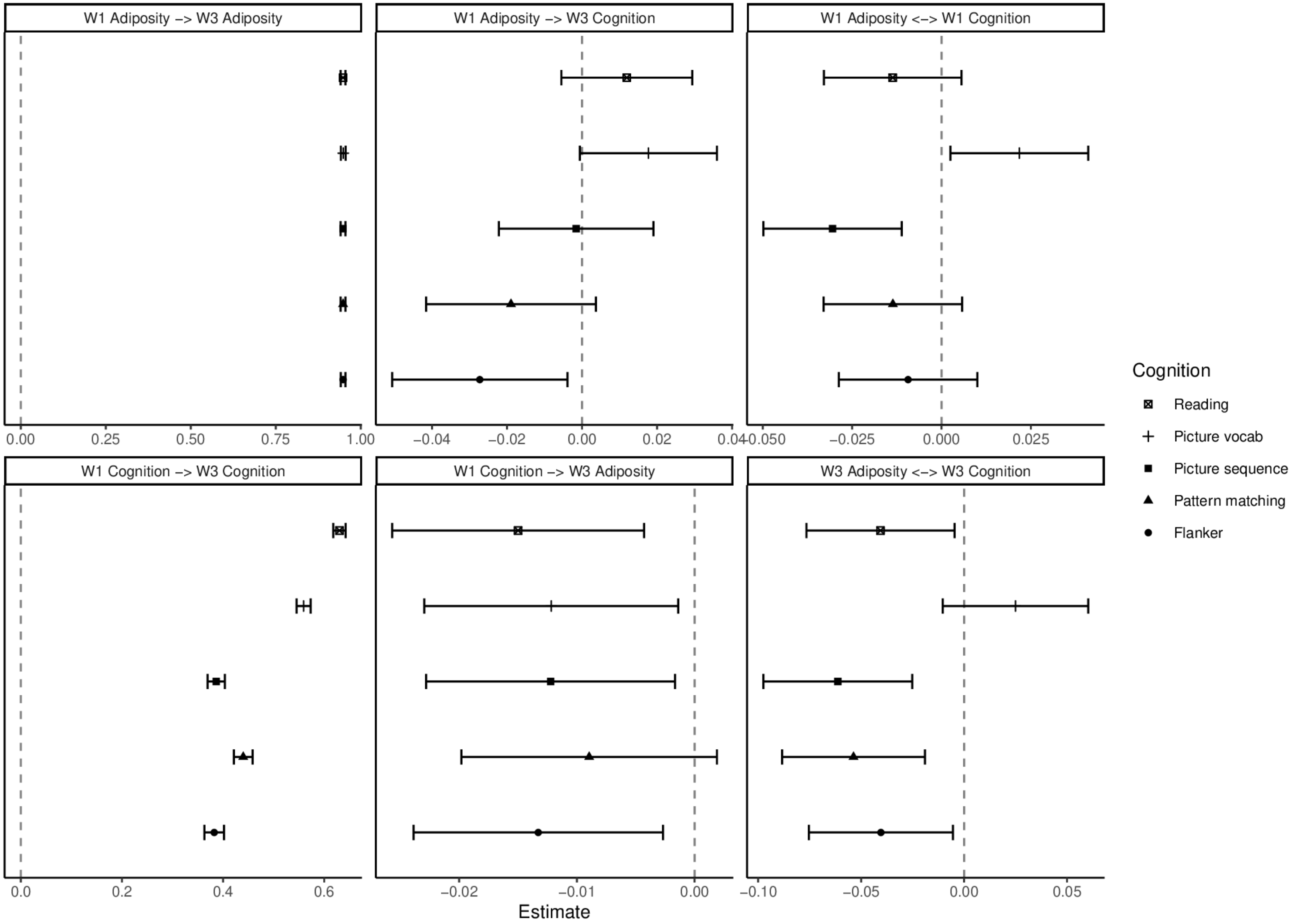

Note: Sensitivity analysis was conducted including study sites and excluding pubertal status and sleep duration as covariates. W1 = Wave 1 or baseline measures; W3 = Wave 3 or 2-year follow-up measures; Adiposity = Latent adiposity variable.

© 2023 Sakib MN et al. *JAMA Network Open.*

eTable 1. Description of Study Sites

Site ID	No. (%)
**Site 01**	367 (3.3%)
**Site 02**	539 (4.9%)
**Site 03**	599 (5.4%)
**Site 04**	725 (6.5%)
**Site 05**	368 (3.3%)
**Site 06**	520 (4.7%)
**Site 07**	326 (2.9%)
**Site 08**	326 (2.9%)
**Site 09**	406 (3.7%)
**Site 10**	705 (6.3%)
**Site 11**	441 (4.0%)
**Site 12**	599 (5.4%)
**Site 13**	636 (5.7%)
**Site 14**	603 (5.4%)
**Site 15**	384 (3.5%)
**Site 16**	996 (9.0%)
**Site 17**	507 (4.6%)
**Site 18**	376 (3.4%)
**Site 19**	447 (4.0%)
**Site 20**	673 (6.1%)
**Site 21**	528 (4.8%)
**Site 22**	32 (0.3%)

Note: The ABCD Research Consortium consists of a Coordinating Center, a Data Analysis and Informatics Center, and 21 research sites across the United States. More information about Study Sites is available at the following link: https://abcdstudy.org/study-sites/.

© 2023 Sakib MN et al. *JAMA Network Open.*

eTable 2. Pearson Product-Moment Correlations among cognitive variables

Measures	1	2	3	4	5	6	7	8	9	10
**Baseline**										
**1. Flanker$_{W1}$**	1.00									
**2. Pattern$_{W1}$**	0.32	1.00								
**3. PicSeq$_{W1}$**	0.17	0.14	1.00							
**4. PicVocab$_{W1}$**	0.17	0.09	0.16	1.00						
**5. Reading$_{W1}$**	0.18	0.12	0.16	0.41	1.00					
**Follow-up**										
**6. Flanker$_{W3}$**	0.38	0.19	0.12	0.16	0.18	1.00				
**7. Pattern$_{W3}$**	0.22	0.45	0.16	0.10	0.13	0.36	1.00			
**8. PicSeq$_{W3}$**	0.13	0.13	0.40	0.13	0.12	0.13	0.18	1.00		
**9. PicVocab$_{W3}$**	0.14	0.09	0.14	0.58	0.41	0.20	0.11	0.18	1.00	
**10. Reading$_{W3}$**	0.13	0.08	0.12	0.39	0.65	0.18	0.14	0.15	0.49	1.00

Note: Flanker = Flanker task; Pattern = Pattern comparison processing speed test; PicSeq = Picture sequence memory test; PicVocab = Picture vocabulary task; Reading = Oral reading Recognition Task; Adiposity = Latent adiposity variable. W1 and W3 indicate Wave 1 (baseline) and Wave 3 or follow-up measures, respectively.

© 2023 Sakib MN et al. *JAMA Network Open.*

eTable 3. Pearson Product-Moment Correlations among adiposity variables

Measures	1	2	3	4
**1. $zBMI_{W1}$**	1.00			
**2. WC_{W1}**	0.85	1.00		
**3. $zBMI_{W3}$**	0.87	0.78	1.00	
**4. WC_{W3}**	0.79	0.79	0.86	1.00

Note: zBMI = BMI z scores; WC = Waist Circumference; W1 = Wave 1 or baseline measures; W3 = Wave 3 or follow-up measures.

© 2023 Sakib MN et al. *JAMA Network Open.*

eTable 4: Sensitivity analysis by removing winsorization and excluding underweight individuals

**Path label**	**Path description**	**Estimate (95% CI)**	***p* value**
		**Flanker Task**	
**a**	$Cog_{w1} \rightarrow Cog_{w3}$	**0.39 (0.37, 0.41)**	**<0.001**
**b**	$Adi_{w1} \rightarrow Cog_{w3}$	**-0.03 (-0.05, 0.00)**	**0.019**
**c**	$Cog_{w1} \rightarrow Adi_{w3}$	**-0.01 (-0.02, 0.00)**	**0.018**
**d**	$Adi_{w1} \rightarrow Adi_{w3}$	**0.93 (0.93, 0.94)**	**<0.001**
**e**	$Adi_{w1} \leftrightarrow Cog_{w1}$	**-0.03 (-0.05, -0.01)**	**0.007**
**f**	$Adi_{w3} \leftrightarrow Cog_{w3}$	**-0.04 (-0.08, -0.01)**	**0.013**
		**Pattern Comparison**	
**a**	$Cog_{w1} \rightarrow Cog_{w3}$	**0.38 (0.37, 0.40)**	**<0.001**
**b**	$Adi_{w1} \rightarrow Cog_{w3}$	**0.00 (-0.03, 0.02)**	**0.041**
**c**	$Cog_{w1} \rightarrow Adi_{w3}$	-0.01 (-0.02, 0.00)	0.302
**d**	$Adi_{w1} \rightarrow Adi_{w3}$	**0.93 (0.93, 0.94)**	**<0.001**
**e**	$Adi_{w1} \leftrightarrow Cog_{w1}$	-0.03 (-0.05, -0.02)	0.109
**f**	$Adi_{w3} \leftrightarrow Cog_{w3}$	**-0.05 (-0.08, -0.01)**	**0.011**
		**Picture Sequence**	
**a**	$Cog_{w1} \rightarrow Cog_{w3}$	**0.44 (0.42, 0.46)**	**<0.001**
**b**	$Adi_{w1} \rightarrow Cog_{w3}$	-0.02 (-0.05, 0.00)	0.672
**c**	$Cog_{w1} \rightarrow Adi_{w3}$	**-0.01 (-0.02, 0.01)**	**0.049**
**d**	$Adi_{w1} \rightarrow Adi_{w3}$	**0.93 (0.93, 0.94)**	**<0.001**
**e**	$Adi_{w1} \leftrightarrow Cog_{w1}$	**-0.02 (-0.04, 0.00)**	**0.001**
**f**	$Adi_{w3} \leftrightarrow Cog_{w3}$	**-0.04 (-0.08, -0.01)**	**0.007**
		**Picture Vocabulary**	
**a**	$Cog_{w1} \rightarrow Cog_{w3}$	**0.56 (0.54, 0.57)**	**<0.001**
**b**	$Adi_{w1} \rightarrow Cog_{w3}$	0.01 (-0.01, 0.03)	0.180
**c**	$Cog_{w1} \rightarrow Adi_{w3}$	**-0.02 (-0.03, 0.00)**	**0.008**
**d**	$Adi_{w1} \rightarrow Adi_{w3}$	**0.93 (0.93, 0.94)**	**<0.001**
**e**	$Adi_{w1} \leftrightarrow Cog_{w1}$	**0.03 (0.01, 0.05)**	**0.007**

© 2023 Sakib MN et al. *JAMA Network Open.*

**f**	$Adi_{W3} \leftrightarrow Cog_{W3}$	0.03 (0.00, 0.06)	0.075
**Path label**	**Path description**	**Estimate (95% CI)**	***p* value**
		**Oral Reading Recognition**	
**a**	$Cog_{W1} \rightarrow Cog_{W3}$	**0.63 (0.62, 0.64)**	**<0.001**
**b**	$Adi_{W1} \rightarrow Cog_{W3}$	0.00 (-0.02, 0.02)	0.777
**c**	$Cog_{W1} \rightarrow Adi_{W3}$	**-0.02 (-0.03, -0.01)**	**<0.001**
**d**	$Adi_{W1} \rightarrow Adi_{W3}$	**0.93 (0.93, 0.94)**	**<0.001**
**e**	$Adi_{W1} \leftrightarrow Cog_{W1}$	-0.01 (-0.03, 0.01)	0.199
**f**	$Adi_{W3} \leftrightarrow Cog_{W3}$	-0.03 (-0.07, 0.00)	0.074

Note 1: Cog_{W1} and Cog_{W3} indicate cognition at baseline (Wave 1) and follow-up (Wave 3), respectively. Adi_{W1} and Adi_{W3} indicate latent adiposity variable at baseline (Wave 1) and follow-up (Wave 1), respectively. Higher scores on cognitive tasks indicate better cognitive status whereas higher scores on adiposity measure indicate worse adiposity status. The analyses were adjusted for age, sex, ethnicity, family income, parent education, area deprivation index, pubertal status and sleep duration. All estimates are standardized coefficients.

Note 2: Description of the cross-lagged paths: (a) Path a: the association between baseline cognition and follow-up cognition, (b) Path b: the association between baseline adiposity and follow-up cognition, (c) Path c: the association between baseline cognition and follow-up adiposity, and (d) Path d: the association between baseline adiposity and follow-up adiposity, € Path e: Covariance between baseline BMI and baseline cognition, and (f) Path f: Covariance between follow-up BMI and follow-up cognition.

© 2023 Sakib MN et al. *JAMA Network Open.*

eTable 5: Sensitivity analysis by including study sites and excluding pubertal status and sleep duration.

Path label	Path description	Estimate (95% CI)	*p* value
		**Flanker Task**	
**a**	$Cog_{w1} \rightarrow Cog_{w3}$	**0.38 (0.36, 0.40)**	**<0.001**
**b**	$Adi_{w1} \rightarrow Cog_{w3}$	**-0.03 (-0.05, -0.01)**	**0.017**
**c**	$Cog_{w1} \rightarrow Adi_{w3}$	**-0.01 (-0.02, 0.00)**	**0.019**
**d**	$Adi_{w1} \rightarrow Adi_{w3}$	**0.95 (0.94, 0.96)**	**<0.001**
**e**	$Adi_{w1} \leftrightarrow Cog_{w1}$	0.00 (-0.02, 0.01)	0.641
**f**	$Adi_{w3} \leftrightarrow Cog_{w3}$	**-0.04 (-0.08, -0.01)**	**0.022**
		**Pattern Comparison**	
**a**	$Cog_{w1} \rightarrow Cog_{w3}$	**0.44 (0.42, 0.46)**	**<0.001**
**b**	$Adi_{w1} \rightarrow Cog_{w3}$	-0.02 (-0.04, 0.00)	0.089
**c**	$Cog_{w1} \rightarrow Adi_{w3}$	-0.01 (-0.02, 0.00)	0.122
**d**	$Adi_{w1} \rightarrow Adi_{w3}$	**0.95 (0.94, 0.96)**	**<0.001**
**e**	$Adi_{w1} \leftrightarrow Cog_{w1}$	-0.01 (-0.03, 0.01)	0.162
**f**	$Adi_{w3} \leftrightarrow Cog_{w3}$	**-0.05 (-0.09, -0.02)**	**0.003**
		**Picture Sequence**	
**a**	$Cog_{w1} \rightarrow Cog_{w3}$	**0.39 (0.37, 0.40)**	**<0.001**
**b**	$Adi_{w1} \rightarrow Cog_{w3}$	-0.01 (-0.03, 0.01)	0.511
**c**	$Cog_{w1} \rightarrow Adi_{w3}$	**-0.01 (-0.02, 0.00)**	**0.024**
**d**	$Adi_{w1} \rightarrow Adi_{w3}$	**0.95 (0.94, 0.96)**	**<0.001**
**e**	$Adi_{w1} \leftrightarrow Cog_{w1}$	**-0.03 (-0.05, -0.01)**	**0.001**
**f**	$Adi_{w3} \leftrightarrow Cog_{w3}$	**-0.06 (-0.10, -0.03)**	**0.001**
		**Picture Vocabulary**	
**a**	$Cog_{w1} \rightarrow Cog_{w3}$	**0.56 (0.55, 0.58)**	**<0.001**
**b**	$Adi_{w1} \rightarrow Cog_{w3}$	0.01 (-0.01, 0.03)	0.174
**c**	$Cog_{w1} \rightarrow Adi_{w3}$	**-0.01 (-0.02, 0.00)**	**0.027**
**d**	$Adi_{w1} \rightarrow Adi_{w3}$	**0.95 (0.94, 0.96)**	**<0.001**
**e**	$Adi_{w1} \leftrightarrow Cog_{w1}$	0.02 (0.00, 0.04)	0.078

© 2023 Sakib MN et al. *JAMA Network Open.*

**f**	$Adi_{W3} \leftrightarrow Cog_{W3}$	0.03 (-0.01, 0.06)	0.165
**Path label**	**Path description**	**Estimate (95% CI)**	***p* value**
		**Oral Reading Recognition**	
**a**	$Cog_{W1} \rightarrow Cog_{W3}$	**0.63 (0.62, 0.64)**	**<0.001**
**b**	$Adi_{W1} \rightarrow Cog_{W3}$	0.01 (-0.01, 0.03)	0.300
**c**	$Cog_{W1} \rightarrow Adi_{W3}$	**-0.01 (-0.03, 0.00)**	**0.008**
**d**	$Adi_{W1} \rightarrow Adi_{W3}$	**0.95 (0.94, 0.96)**	**<0.001**
**e**	$Adi_{W1} \leftrightarrow Cog_{W1}$	**-0.02 (-0.04, 0.00)**	**0.048**
**f**	$Adi_{W3} \leftrightarrow Cog_{W3}$	**-0.04 (-0.08, 0.00)**	**0.027**

Note 1: Cog_{W1} and Cog_{W3} indicate cognition at baseline (Wave 1) and follow-up (Wave 3), respectively. Adi_{W1} and Adi_{W3} indicate latent adiposity variable at baseline (Wave 1) and follow-up (Wave 1), respectively. Higher scores on cognitive tasks indicate better cognitive status whereas higher scores on adiposity measure indicate worse adiposity status. The analyses were adjusted for age, sex, ethnicity, family income, parent education, area deprivation index, pubertal status and sleep duration. All estimates are standardized coefficients.

Note 2: Description of the cross-lagged paths: (a) Path a: the association between baseline cognition and follow-up cognition, (b) Path b: the association between baseline adiposity and follow-up cognition, (c) Path c: the association between baseline cognition and follow-up adiposity, and (d) Path d: the association between baseline adiposity and follow-up adiposity, € Path e: Covariance between baseline BMI and baseline cognition, and (f) Path f: Covariance between follow-up BMI and follow-up cognition.

© 2023 Sakib MN et al. *JAMA Network Open.*

eTable 6. Mediation analysis for the path 2 (Adiposity → Mediator → Cognition)

Mediators	Indirect effect (95% CI)	*p*	Indirect effect (95% CI)	*p*
	$zBMI_{W1}$ → Mediator → $Flanker_{W3}$		WC_{W1} → Mediator → $Flanker_{W3}$	
**Physical activity**	-0.0145 (-0.0360, 0.0069)	0.185	-0.0074 (-0.0175, 0.0027)	0.150
**Diet**				
**Total**	-0.0016 (-0.0079, 0.0047)	0.621	-0.0015 (-0.0051, 0.0022)	0.433
**Whole grains**	-0.0001 (-0.0033, 0.0031)	0.947	-0.0001 (-0.0021, 0.0019)	0.940
**Green leafy vegetables**	0.0008 (-0.0018, 0.0033)	0.540	0.0003 (-0.0011, 0.0018)	0.647
**Other vegetables**	-0.0006 (-0.0020, 0.0009)	0.441	-0.0005 (-0.0015, 0.0005)	0.364
**Berries**	-0.0001 (-0.0013, 0.0012)	0.926	-0.0001 (-0.0018, 0.0017)	0.936
**Beans**	-0.0012 (-0.0035, 0.0011)	0.311	-0.0005 (-0.0015, 0.0005)	0.351
**Nuts**	-0.0001 (-0.0013, 0.0011)	0.901	-0.0002 (-0.0010, 0.0005)	0.533
**Fast/fried food**	-0.0010 (-0.0052, 0.0032)	0.645	-0.0006 (-0.0027, 0.0016)	0.607
**Pastries or sweets**	0.0006 (-0.0008, 0.0020)	0.419	0.0001 (-0.0005, 0.0007)	0.687
**Blood pressure**				
**Total**	0.0305 (-0.0062, 0.0672)	0.103	0.0182 (-0.0031, 0.0396)	0.095
**Systolic**	0.0370 (-0.0045, 0.0785)	0.081	0.0226 (-0.0020, 0.0473)	0.072
**Diastolic**	-0.0065 (-0.0470, 0.0341)	0.755	-0.0044 (-0.0273, 0.0185)	0.705
**LPFC volume**				
**Total**	-0.0064 (-0.0132, 0.0004)	0.066	-0.0049 (-0.0093, -0.0005)	0.029
**LOFC**	-0.0017 (-0.0048, 0.0014)	0.271	-0.0016 (-0.0042, 0.0010)	0.227
**MFG**	-0.0036 (-0.0112, 0.0039)	0.346	-0.0026 (-0.0077, 0.0026)	0.328
**IFG**	-0.0010 (-0.0050, 0.0030)	0.625	-0.0007 (-0.0037, 0.0023)	0.632
**LPFC thickness**				
**Total**	0.0017 (-0.0130, 0.0165)	0.817	0.0006 (-0.0046, 0.0058)	0.815
**Lateral OFC**	-0.0026 (-0.0203, 0.0152)	0.776	-0.0010 (-0.0074, 0.0054)	0.757
**MFG**	0.0018 (-0.0140, 0.0176)	0.823	0.0004 (-0.0046, 0.0054)	0.873
**IFG**	0.0025 (-0.0116, 0.0166)	0.726	0.0012 (-0.0048, 0.0072)	0.694
	$zBMI_{W1}$ → Mediator → $Pattern_{W3}$		WC_{W1} → Mediator → $Pattern_{W3}$	
**Physical activity**	**-0.0387 (-0.0687, -0.0086)**	**0.012**	**-0.0192 (-0.0334, -0.005)**	**0.008**
**Diet**				

© 2023 Sakib MN et al. *JAMA Network Open.*

**Total**	0.0034 (-0.0050, 0.0119)	0.428	0.0008 (-0.0041, 0.0057)	0.748
**Mediators**	**Indirect effect (95% CI)**	***p***	**Indirect effect (95% CI)**	***p***
**Whole grains**	-0.0018 (-0.0064, 0.0027)	0.434	-0.0012 (-0.0040, 0.0017)	0.430
**Green leafy vegetables**	0.0003 (-0.0010, 0.0017)	0.617	0.0002 (-0.0006, 0.0009)	0.678
**Other vegetables**	0.0012 (-0.0014, 0.0037)	0.371	0.0010 (-0.0007, 0.0027)	0.251
**Berries**	-0.0009 (-0.0029, 0.0012)	0.426	-0.0012 (-0.0037, 0.0013)	0.348
**Beans**	-0.0002 (-0.0029, 0.0026)	0.916	-0.0001 (-0.0012, 0.0011)	0.898
**Nuts**	-0.0001 (-0.0012, 0.0011)	0.913	-0.0002 (-0.0011, 0.0006)	0.583
**Fast/fried food**	0.0047 (-0.0015, 0.0108)	0.135	0.0023 (-0.0008, 0.0055)	0.151
**Pastries or sweets**	0.0001 (-0.0013, 0.0016)	0.862	0.0000 (-0.0003, 0.0004)	0.879
**Blood pressure**				
**Total**	-0.0109 (-0.0620, 0.0402)	0.677	-0.0044 (-0.0342, 0.0255)	0.774
**Systolic**	0.0553 (-0.0023, 0.1129)	0.060	0.0332 (-0.0012, 0.0676)	0.058
**Diastolic**	-0.0662 (-0.1229, -0.0094)	0.022	-0.0376 (-0.0697, -0.0055)	0.022
**LPFC volume**				
**Total**	0.0077 (-0.0013, 0.0166)	0.094	0.0051 (-0.0007, 0.0110)	0.086
**LOFC**	-0.0006 (-0.0042, 0.0030)	0.745	-0.0006 (-0.0039, 0.0028)	0.748
**MFG**	0.0075 (-0.0032, 0.0181)	0.170	0.0051 (-0.0022, 0.0123)	0.169
**IFG**	0.0008 (-0.0047, 0.0063)	0.776	0.0006 (-0.0035, 0.0048)	0.763
**LPFC thickness**				
**Total**	0.0200 (-0.0021 0.0420)	0.076	0.0045 (-0.0042, 0.0131)	0.310
**LOFC**	-0.0005 (-0.0253, 0.0243)	0.971	-0.0004 (-0.0093, 0.0085)	0.921
**MFG**	**0.0373 (0.0143, 0.0602)**	**0.001**	**0.0117 (0.0036, 0.0199)**	**0.005**
**IFG**	-0.0168 (-0.0370, 0.0034)	0.102	-0.0068 (-0.0156, 0.0020)	0.130
	**$zBMI_{W1}$ → Mediator → $PicSeq_{W3}$**		**WC_{W1} → Mediator → $PicSeq_{W3}$**	
**Physical activity**	0.0002 (-0.0249, 0.0254)	0.985	0.0002 (-0.0116, 0.0119)	0.977
**Diet**				
**Total**	-0.0031 (-0.0099, 0.0036)	0.360	-0.0013 (-0.0052, 0.0026)	0.513
**Whole grains**	-0.0004 (-0.0038, 0.0030)	0.807	-0.0002 (-0.0024, 0.0019)	0.829
**Green leafy vegetables**	0.0006 (-0.0013, 0.0025)	0.537	0.0003 (-0.0008, 0.0013)	0.640
**Other vegetables**	-0.0007 (-0.0024, 0.0010)	0.408	-0.0006 (-0.0018, 0.0006)	0.295
**Berries**	0.0000 (-0.0012, 0.0013)	0.955	0.0001 (-0.0018, 0.0019)	0.941

© 2023 Sakib MN et al. *JAMA Network Open.*

**Beans**	0.0001 (-0.0021, 0.0023)	0.941	0.0000 (-0.0009, 0.0009)	0.945
**Nuts**	0.0001 (-0.0020, 0.0023)	0.896	0.0004 (-0.0008, 0.0017)	0.496
**Mediators**	**Indirect effect (95% CI)**	***p***	**Indirect effect (95% CI)**	***p***
**Fast/fried food**	-0.0022 (-0.0067, 0.0024)	0.347	-0.0011 (-0.0034, 0.0012)	0.357
**Pastries or sweets**	-0.0007 (-0.0023, 0.0009)	0.399	-0.0001 (-0.0008, 0.0005)	0.678
**Blood pressure**				
**Total**	-0.0339 (-0.0743, 0.0065)	0.100	-0.0170 (-0.0405, 0.0064)	0.155
**Systolic**	0.0088 (-0.0364, 0.0541)	0.702	0.0064 (-0.0205, 0.0333)	0.642
**Diastolic**	-0.0428 (-0.0876, 0.0021)	0.062	-0.0234 (-0.0487, 0.0019)	0.070
**LPFC volume**				
**Total**	0.0004 (-0.0070, 0.0078)	0.915	0.0000 (-0.0048, 0.0049)	0.993
**LOFC**	-0.0009 (-0.0040, 0.0022)	0.575	-0.0009 (-0.0037, 0.0020)	0.555
**MFG**	0.0019 (-0.0069, 0.0107)	0.669	0.0013 (-0.0046, 0.0073)	0.659
**IFG**	-0.0006 (-0.0053, 0.0041)	0.793	-0.0005 (-0.0040, 0.0031)	0.796
**LPFC thickness**				
**Total**	-0.0046 (-0.0224, 0.0132)	0.615	-0.0011 (-0.0075, 0.0053)	0.735
**LOFC**	0.0129 (-0.0089, 0.0346)	0.246	0.0053 (-0.0026, 0.0132)	0.186
**MFG**	-0.0125 (-0.0326, 0.0075)	0.221	-0.0037 (-0.0102, 0.0029)	0.270
**IFG**	-0.0049 (-0.0230, 0.0131)	0.593	-0.0027 (-0.0105, 0.0051)	0.492
	**zBMI$_{W1}$ → Mediator → PicVocab$_{W3}$**		**WC$_{W1}$ → Mediator → PicVocab$_{W3}$**	
**Physical activity**	**-0.0489 (-0.0692, -0.0286)**	**<0.001**	**-0.0224 (-0.0321, -0.0127)**	**<0.000**
**Diet**				
**Total**	-0.0005 (-0.0057, 0.0048	0.865	0.0002 (-0.0029, 0.0032)	0.907
**Whole grains**	0.0009 (-0.0018, 0.0035)	0.528	0.0005 (-0.0012, 0.0022)	0.543
**Green leafy vegetables**	0.0002 (-0.0007, 0.0012)	0.614	0.0001 (-0.0004, 0.0006)	0.707
**Other vegetables**	-0.0010 (-0.0031, 0.0010)	0.330	-0.0009 (-0.0022, 0.0004)	0.183
**Berries**	0.0009 (-0.0008, 0.0026)	0.292	0.0013 (-0.0003, 0.0030)	0.115
**Beans**	0.0000 (-0.0017, 0.0016)	0.973	0.0000 (-0.0007, 0.0007)	0.997
**Nuts**	-0.0001 (-0.0010, 0.0008)	0.902	-0.0002 (-0.0008, 0.0004)	0.538
**Fast/fried food**	-0.0014 (-0.0050, 0.0022)	0.458	-0.0007 (-0.0026, 0.0012)	0.467
**Pastries or sweets**	0.0000 (-0.0009, 0.0009)	0.972	0.0000 (-0.0002, 0.0002)	0.989
**Blood pressure**				

© 2023 Sakib MN et al. *JAMA Network Open.*

Total	-0.0106 (-0.0424, 0.0213)	0.515	-0.0094 (-0.0279, 0.0091)	0.319
Systolic	-0.0081 (-0.0441, 0.0279)	0.659	-0.0075 (-0.0289, 0.0139)	0.490
Diastolic	-0.0025 (-0.0379, 0.0329)	0.891	-0.0019 (-0.0218, 0.0181)	0.854
**Mediators**	**Indirect effect (95% CI)**	***p***	**Indirect effect (95% CI)**	***p***
**LPFC volume**				
Total	-0.0127 (-0.0204, -0.0050)	0.001	-0.0096 (-0.0144, -0.0049)	0.000
LOFC	-0.0027 (-0.0063, 0.0008)	0.129	-0.0026 (-0.0053, 0.0001)	0.059
MFG	-0.0068 (-0.0139, 0.0004)	0.063	-0.0047 (-0.0095, 0.0001)	0.057
IFG	-0.0032 (-0.0073, 0.0010)	0.136	-0.0024 (-0.0053, 0.0006)	0.117
**LPFC thickness**				
Total	0.0096 (-0.0047, 0.0239)	0.189	0.0054 (-0.0003, 0.0111)	0.062
LOFC	0.0137 (-0.0024, 0.0298)	0.096	0.0050 (-0.0008, 0.0109)	0.091
MFG	**-0.0183 (-0.0331, -0.0035)**	**0.015**	**-0.0061 (-0.0113, -0.0009)**	**0.021**
IFG	**0.0142 (0.0012, 0.0272)**	**0.033**	**0.0065 (0.0007, 0.0123)**	**0.029**
	**$zBMI_{W1}$ → Mediator → $Reading_{W3}$**		**WC_{W1} → Mediator → $Reading_{W3}$**	
**Physical activity**	**-0.0214 (-0.0406, -0.0022)**	**0.029**	**-0.0108 (-0.0199, -0.0017)**	**0.020**
**Diet**				
Total	0.0000 (-0.0050, 0.0051)	0.986	0.0003 (-0.0026, 0.0033)	0.820
Whole grains	0.0009 (-0.0018, 0.0035)	0.531	0.0005 (-0.0012, 0.0022)	0.549
Green leafy vegetables	0.0004 (-0.0009, 0.0018)	0.546	0.0002 (-0.0006, 0.0009)	0.667
Other vegetables	-0.0004 (-0.0015, 0.0007)	0.479	-0.0003 (-0.0011, 0.0005)	0.412
Berries	0.0004 (-0.0008, 0.0016)	0.489	0.0006 (-0.0009, 0.0020)	0.462
Beans	0.0007 (-0.0011, 0.0025)	0.457	0.0003 (-0.0005, 0.0010)	0.487
Nuts	0.0000 (-0.0006, 0.0007)	0.890	0.0001 (-0.0004, 0.0006)	0.582
Fast/fried food	-0.0017 (-0.0052, 0.0018)	0.343	-0.0009 (-0.0027, 0.0009)	0.330
Pastries or sweets	-0.0003 (-0.0013, 0.0007)	0.578	-0.0001 (-0.0004, 0.0003)	0.725
**Blood pressure**				
Total	-0.0185 (-0.0504, 0.0133)	0.253	-0.0135 (-0.0319, 0.0050)	0.153
Systolic	**-0.0408 (-0.0768, -0.0048)**	**0.026**	**-0.0256 (-0.0470, -0.0042)**	**0.019**
Diastolic	0.0223 (-0.0130, 0.0575)	0.215	0.0121 (-0.0077, 0.0320)	0.231
**LPFC volume**				
Total	**-0.0109 (-0.0179, -0.0040)**	**0.002**	**-0.0082 (-0.0126, -0.0038)**	**<0.001**

© 2023 Sakib MN et al. *JAMA Network Open.*

**LOFC**	-0.0020 (-0.0049, 0.0010)	0.196	-0.0018 (-0.0043, 0.0006)	0.131
**MFG**	**-0.0073 (-0.0143, -0.0003)**	**0.041**	**-0.0051 (-0.0099, -0.0004)**	**0.034**
**IFG**	-0.0016 (-0.0053, 0.0020)	0.374	-0.0013 (-0.0040, 0.0015)	0.364
**Mediators**	**Indirect effect (95% CI)**	***p***	**Indirect effect (95% CI)**	***p***
**LPFC thickness**				
**Total**	-0.0052 (-0.0186, 0.0082)	0.450	-0.0024 (-0.0071, 0.0023)	0.317
**LOFC**	-0.0029 (-0.0193, 0.0134)	0.726	-0.0012 (-0.0071, 0.0046)	0.677
**MFG**	0.0017 (-0.0130, 0.0165)	0.818	0.0006 (-0.0042, 0.0053)	0.816
**IFG**	-0.0040 (-0.0172, 0.0093)	0.556	-0.0017 (-0.0074, 0.0040)	0.555

Note: LPFC = lateral prefrontal cortex; LOFC = lateral orbitofrontal cortex; MFG = middle frontal gyrus; IFG = inferior frontal gyrus. Path b: the association between baseline adiposity and follow-up cognition. Four decimal places were retained because of the smaller values of the indirect effects and to clarify the direction of coefficient and CI. Total indicates the sum of individual indirect effects for the respective mediators. All estimates are unstandardized coefficients. Significant indirect effects indicate that the association between adiposity and cognition is, in part, mediated through that respective variable.

© 2023 Sakib MN et al. *JAMA Network Open.*

eTable 7. Mediation analysis for path 3 (Cognition → Mediator → Adiposity)

Mediators	Indirect effect (95% CI)	*p*	Indirect effect (95% CI)	*p*
	Flanker$_{W1}$ → Mediator → zBMI$_{W3}$		Flanker$_{W1}$ → Mediator → WC$_{W3}$	
**Physical activity**	0.0000 (-0.0004, 0.0004)	0.902	-0.0001 (-0.0008, 0.0007)	0.860
**Diet**				
**Total**	0.0001 (-0.0001, 0.0003)	0.175	0.0005 (0.0000, 0.0009)	0.043
**Whole grains**	0.0000 (0.0000, 0.0000)	0.765	0.0000 (-0.0001, 0.0001)	0.659
**Green leafy vegetables**	0.0000 (0.0000, 0.0001)	0.399	0.0002 (-0.0001, 0.0004)	0.142
**Other vegetables**	0.0000 (-0.0001, 0.0001)	0.675	0.0000 (-0.0002, 0.0002)	0.851
**Berries**	0.0000 (0.0000, 0.0001)	0.335	0.0000 (-0.0001, 0.0001)	0.758
**Beans**	0.0000 (-0.0001, 0.0001)	0.473	0.0002 (-0.0001, 0.0004)	0.149
**Nuts**	0.0000 (-0.0001, 0.0000)	0.449	0.0000 (-0.0002, 0.0001)	0.461
**Fast/fried food**	0.0001 (0.0000, 0.0002)	0.145	0.0002 (-0.0001, 0.0005)	0.128
**Pastries or sweets**	0.0000 (-0.0001, 0.0000)	0.402	0.0000 (-0.0001, 0.0001)	0.569
**LPFC volume**				
**Total**	0.0000 (-0.0001, 0.0001)	0.693	0.0000 (-0.0002, 0.0001)	0.661
**LOFC**	0.0000 (-0.0001, 0.0000)	0.703	0.0000 (-0.0001, 0.0001)	0.731
**MFG**	0.0000 (0.0000, 0.0000)	0.987	0.0000 (-0.0001, 0.0001)	0.985
**IFG**	0.0000 (0.0000, 0.0000)	0.819	0.0000 (-0.0001, 0.0001)	0.802
**LPFC thickness**				
**Total**	0.0000 (-0.0001, 0.0001)	0.909	-0.0002 (-0.0006, 0.0002)	0.390
**LOFC**	0.0000 (0.0000, 0.0001)	0.374	0.0000 (-0.0001, 0.0002)	0.690
**MFG**	0.0000 (-0.0001, 0.0001)	0.533	-0.0002 (-0.0005, 0.0002)	0.269
**IFG**	0.0000 (-0.0001, 0.0001)	0.863	0.0000 (-0.0001, 0.0001)	0.825
	Pattern$_{W1}$ → Mediator → zBMI$_{W3}$		Pattern$_{W1}$ → Mediator → WC$_{W3}$	
**Physical activity**	0.0000 (-0.0003, 0.0003)	0.989	0.0000 (-0.0005, 0.0005)	0.928
**Diet**				
**Total**	0.0000 (-0.0002, 0.0001)	0.413	0.0000 (-0.0003, 0.0003)	0.902
**Whole grains**	0.0000 (0.0000, 0.0000)	0.752	0.0000 (0.0000, 0.0001)	0.599
**Green leafy vegetables**	0.0000 (0.0000, 0.0001)	0.401	0.0001 (0.0000, 0.0003)	0.129
**Other vegetables**	0.0000 (0.0000, 0.0000)	0.712	0.0000 (-0.0001, 0.0001)	0.809

© 2023 Sakib MN et al. *JAMA Network Open.*

**Berries**	0.0000 (0.0000, 0.0000)	0.856	0.0000 (0.0000, 0.0000)	0.887
**Mediators**	**Indirect effect (95% CI)**	***p***	**Indirect effect (95% CI)**	***p***
**Beans**	0.0000 (0.0000, 0.0000)	0.796	0.0000 (-0.0001, 0.0001)	0.786
**Nuts**	0.0000 (0.0000, 0.0000)	0.757	0.0000 (-0.0001, 0.0001)	0.755
**Fast/fried food**	0.0000 (-0.0001, 0.0000)	0.198	-0.0001 (-0.0003, 0.0000)	0.180
**Pastries or sweets**	0.0000 (-0.0001, 0.0000)	0.170	0.0000 (-0.0001, 0.0001)	0.464
**LPFC volume**				
**Total**	0.0000 (-0.0001, 0.0001)	0.740	0.0000 (-0.0002, 0.0002)	0.974
**LOFC**	0.0000 (0.0000, 0.0000)	0.534	0.0000 (-0.0001, 0.0001)	0.555
**MFG**	0.0000 (0.0000, 0.0001)	0.268	0.0001 (-0.0001, 0.0002)	0.421
**IFG**	0.0000 (-0.0001, 0.0000)	0.479	0.0000 (-0.0002, 0.0001)	0.402
**LPFC thickness**				
**Total**	0.0000 (-0.0001, 0.0002)	0.561	**0.0004 (0.0001, 0.0006)**	**0.011**
**LOFC**	0.0001 (0.0000, 0.0002)	0.196	0.0001 (-0.0001, 0.0003)	0.262
**MFG**	0.0001 (0.0000, 0.0002)	0.280	0.0002 (-0.0001, 0.0005)	0.117
**IFG**	-0.0001 (-0.0002, 0.0000)	0.196	0.0000 (-0.0002, 0.0002)	0.733
	**PicSeq$_{W1}$ → Mediator → zBMI$_{W3}$**		**PicSeq$_{W1}$ → Mediator → WC$_{W3}$**	
**Physical activity**	0.0000 (-0.0003, 0.0003)	0.893	-0.0002 (-0.0008, 0.0004)	0.621
**Diet**				
**Total**	0.0000 (-0.0002, 0.0001)	0.787	0.0001 (-0.0002, 0.0005)	0.482
**Whole grains**	0.0000 (0.0000, 0.0000)	0.918	0.0000 (0.0000, 0.0000)	0.947
**Green leafy vegetables**	0.0000 (0.0000, 0.0001)	0.372	0.0001 (-0.0001, 0.0003)	0.156
**Other vegetables**	0.0000 (0.0000, 0.0000)	0.743	0.0000 (-0.0001, 0.0001)	0.794
**Berries**	0.0000 (0.0000, 0.0000)	0.769	0.0000 (0.0000, 0.0000)	0.842
**Beans**	0.0000 (0.0000, 0.0001)	0.509	0.0001 (-0.0001, 0.0003)	0.214
**Nuts**	0.0000 (-0.0001, 0.0000)	0.490	0.0000 (-0.0001, 0.0001)	0.498
**Fast/fried food**	0.0000 (-0.0001, 0.0000)	0.490	-0.0001 (-0.0003, 0.0001)	0.450
**Pastries or sweets**	0.0000 (-0.0001, 0.0000)	0.391	0.0000 (-0.0001, 0.0001)	0.558
**LPFC volume**				
**Total**	0.0000 (-0.0001, 0.0000)	0.396	-0.0001 (-0.0003, 0.0001)	0.327
**LOFC**	0.0000 (-0.0001, 0.0000)	0.483	0.0000 (-0.0002, 0.0001)	0.508
**MFG**	0.0000 (0.0000, 0.0000)	0.883	0.0000 (-0.0001, 0.0001)	0.821

© 2023 Sakib MN et al. *JAMA Network Open.*

IFG	0.0000 (-0.0001, 0.0000)	0.565	0.0000 (-0.0002, 0.0001)	0.531
**Mediators**	**Indirect effect (95% CI)**	***p***	**Indirect effect (95% CI)**	***p***
**LPFC thickness**				
**Total**	0.0000 (-0.0001, 0.0002)	0.589	0.0004 (0.0001, 0.0008)	0.023
**LOFC**	0.0001 (0.0000, 0.0002)	0.206	0.0002 (-0.0001, 0.0004)	0.248
**MFG**	0.0000 (0.0000, 0.0001)	0.340	0.0002 (-0.0001, 0.0005)	0.168
**IFG**	-0.0001 (-0.0002, 0.0001)	0.225	0.0001 (-0.0003, 0.0004)	0.744
	**$PicVocab_{W1}$ → Mediator → $zBMI_{W3}$**		**$PicVocab_{W1}$ → Mediator → WC_{W3}**	
**Physical activity**	**-0.0011 (-0.0015, -0.0007)**	**<0.001**	**-0.0020 (-0.002, -0.0012)**	**<0.001**
**Diet**				
**Total**	0.0000 (-0.0002, 0.0001)	0.716	-0.0001 (-0.0005, 0.0003)	0.591
**Whole grains**	0.0000 (-0.0001, 0.0000)	0.799	0.0000 (-0.0002, 0.0001)	0.537
**Green leafy vegetables**	0.0000 (0.0000, 0.0001)	0.442	0.0001 (-0.0001, 0.0003)	0.242
**Other vegetables**	0.0000 (0.0000, 0.0000)	0.761	0.0000 (0.0000, 0.0000)	0.804
**Berries**	0.0000 (0.0000, 0.0001)	0.505	0.0000 (0.0000, 0.0001)	0.782
**Beans**	0.0000 (0.0000, 0.0000)	0.655	0.0000 (-0.0001, 0.0001)	0.595
**Nuts**	0.0000 (0.0000, 0.0001)	0.544	0.0000 (-0.0001, 0.0001)	0.539
**Fast/fried food**	-0.0001 (-0.0002, 0.0000)	0.079	-0.0002 (-0.0005, 0.0000)	0.051
**Pastries or sweets**	0.0000 (0.0000, 0.0001)	0.619	0.0000 (0.0000, 0.0001)	0.664
**LPFC volume**				
**Total**	-0.0001 (-0.0002, 0.0000)	0.171	-0.0001 (-0.0004, 0.0001)	0.311
**LOFC**	-0.0001 (-0.0002, 0.0001)	0.389	-0.0001 (-0.0005, 0.0002)	0.419
**MFG**	0.0000 (-0.0001, 0.0000)	0.309	-0.0001 (-0.0003, 0.0001)	0.445
**IFG**	0.0000 (0.0000, 0.0001)	0.468	0.0001 (-0.0001, 0.0002)	0.382
**LPFC thickness**				
**Total**	0.0000 (-0.0001, 0.0001)	0.736	-0.0001 (-0.0005, 0.0002)	0.445
**LOFC**	0.0000 (-0.0001, 0.0001)	0.919	0.0000 (-0.0001, 0.0002)	0.714
**MFG**	0.0000 (-0.0001, 0.0000)	0.362	-0.0001 (-0.0004, 0.0002)	0.341
**IFG**	0.0000 (-0.0001, 0.0001)	0.563	0.0000 (-0.0002, 0.0001)	0.854
	**$Reading_{W1}$ → Mediator → $zBMI_{W3}$**		**$Reading_{W1}$ → Mediator → WC_{W3}**	
**Physical activity**	**-0.0007 (-0.0010, -0.0003)**	**<0.001**	**-0.0013 (-0.0020, -0.0007)**	**<0.001**

© 2023 Sakib MN et al. *JAMA Network Open.*

**Diet**				
**Total**	0.0000 (-0.0001, 0.0002)	0.798	0.0001 (-0.0002, 0.0005)	0.448
**Mediators**	**Indirect effect (95% CI)**	***p***	**Indirect effect (95% CI)**	***p***
**Whole grains**	0.0000 (-0.0001, 0.0001)	0.788	-0.0001 (-0.0002, 0.0001)	0.536
**Green leafy vegetables**	0.0000 (0.0000, 0.0001)	0.369	0.0002 (0.0000, 0.0004)	0.088
**Other vegetables**	0.0000 (0.0000, 0.0000)	0.806	0.0000 (0.0000, 0.0000)	0.814
**Berries**	0.0000 (0.0000, 0.0001)	0.298	0.0000 (-0.0001, 0.0001)	0.734
**Beans**	0.0000 (0.0000, 0.0001)	0.475	0.0001 (0.0000, 0.0003)	0.148
**Nuts**	0.0000 (-0.0001, 0.0000)	0.309	-0.0001 (-0.0002, 0.0001)	0.330
**Fast/fried food**	0.0000 (-0.0001, 0.0000)	0.372	-0.0001 (-0.0003, 0.0001)	0.328
**Pastries or sweets**	0.0000 (-0.0001, 0.0000)	0.784	0.0000 (-0.0001, 0.0000)	0.807
**LPFC volume**				
**Total**	-0.0001 (-0.0001, 0.0000)	0.201	-0.0001 (-0.0003, 0.0001)	0.478
**LOFC**	0.0000 (-0.0001, 0.0001)	0.404	-0.0001 (-0.0003, 0.0001)	0.455
**MFG**	0.0000 (-0.0001, 0.0000)	0.329	-0.0001 (-0.0002, 0.0001)	0.489
**IFG**	0.0000 (0.0000, 0.0001)	0.436	0.0001 (-0.0001, 0.0002)	0.344
**LPFC thickness**				
**Total**	0.0000 (-0.0001, 0.0001)	0.974	0.0000 (-0.0003, 0.0003)	0.996
**LOFC**	0.0000 (0.0000, 0.0001)	0.428	0.0001 (-0.0001, 0.0003)	0.345
**MFG**	0.0000 (-0.0001, 0.0000)	0.545	-0.0001 (-0.0003, 0.0002)	0.568
**IFG**	0.0000 (-0.0001, 0.0001)	0.845	0.0000 (-0.0001, 0.0001)	0.853

Note: LPFC = lateral prefrontal cortex; LOFC = lateral orbitofrontal cortex; MFG = middle frontal gyrus; IFG = inferior frontal gyrus. Path c: the association between baseline cognition and follow-up adiposity. Four decimal places were retained because of the smaller values of the indirect effects and to clarify the direction of coefficient and CI. Total indicates the sum of individual indirect effects for the respective mediators. All estimates are unstandardized coefficients. Significant indirect effects indicate that the association between adiposity and cognition is, in part, mediated through that respective variable.

© 2023 Sakib MN et al. *JAMA Network Open.*

eReferences

1. World Health Organization. *WHO child growth standards: length/height-for-age, weight-for-age, weight-for-length, weight-for-height and body mass index-for-age: methods and development*. World Health Organization; 2006.
2. Myatt M, Guevarra E. zscorer: child anthropometry z-score calculator. R package version 0.3.1. Accessed 11 January, 2022. https://cran.r-project.org/web/packages/zscorer/index.html
3. Eriksen BA, Eriksen CW. Effects of noise letters upon the identification of a target letter in a nonsearch task. *Perception & Psychophysics*. 1974/01/01 1974;16(1):143-149. doi:10.3758/BF03203267
4. Zelazo PD, Anderson JE, Richler J, Wallner-Allen K, Beaumont JL, Weintraub S. NIH toolbox cognition battery (CB): measuring executive function and attention. *Monographs of the Society for Research in Child Development*. 2013;78(4):16-33. doi:https://doi.org/10.1111/mono.12032
5. Luciana M, Bjork JM, Nagel BJ, et al. Adolescent neurocognitive development and impacts of substance use: Overview of the adolescent brain cognitive development (ABCD) baseline neurocognition battery. *Developmental Cognitive Neuroscience*. 2018/08/01/ 2018;32:67-79. doi:https://doi.org/10.1016/j.dcn.2018.02.006
6. Weintraub S, Dikmen SS, Heaton RK, et al. Cognition assessment using the NIH Toolbox. *Neurology*. 2013;80(11 Supplement 3):S54-S64. doi:10.1212/WNL.0b013e3182872ded
7. Carlozzi NE, Tulsky DS, Kail RV, Beaumont JL. NIH Toolbox Cognition Battery (CB): measuring processing speed. *Monogr Soc Res Child Dev*. Aug 2013;78(4):88-102. doi:10.1111/mono.12036
8. Carlozzi NE, Tulsky DS, Chiaravalloti ND, et al. NIH Toolbox Cognitive Battery (NIHTB-CB): the NIHTB Pattern Comparison Processing Speed Test. *J Int Neuropsychol Soc*. Jul 2014;20(6):630-41. doi:10.1017/s1355617714000319
9. Carlozzi NE, Beaumont JL, Tulsky DS, Gershon RC. The NIH Toolbox Pattern Comparison Processing Speed Test: Normative Data. *Arch Clin Neuropsychol*. Aug 2015;30(5):359-68. doi:10.1093/arclin/acv031
10. Bauer PJ, Dikmen SS, Heaton RK, Mungas D, Slotkin J, Beaumont JL. NIH Toolbox Cognition Battery (CB): measuring episodic memory. *Monogr Soc Res Child Dev*. Aug 2013;78(4):34-48. doi:10.1111/mono.12033
11. Dikmen SS, Bauer PJ, Weintraub S, et al. Measuring episodic memory across the lifespan: NIH Toolbox Picture Sequence Memory Test. *J Int Neuropsychol Soc*. Jul 2014;20(6):611-9. doi:10.1017/s1355617714000460
12. Gershon RC, Cook KF, Mungas D, et al. Language Measures of the NIH Toolbox Cognition Battery. *Journal of the International Neuropsychological Society*. 2014;20(6):642-651. doi:10.1017/S1355617714000411
13. Gershon RC, Slotkin J, Manly JJ, et al. NIH toolbox cognition battery (Cb): measuring language (Vocabulary comprehension and reading decoding). Chapter IV. *Monographs of the Society for Research in Child Development*. 2013;78(4):49-69. doi:https://doi.org/10.1111/mono.12034
14. NIMH Data Archive. NDA. Accessed 17 July, 2022. https://nda.nih.gov/data_dictionary.html

© 2023 Sakib MN et al. *JAMA Network Open.*

15. Maroko AR, Doan TM, Arno PS, Hubel M, Yi S, Viola D. Integrating Social Determinants of Health With Treatment and Prevention: A New Tool to Assess Local Area Deprivation. *Prev Chronic Dis*. 2016;13:E128-E128. doi:10.5888/pcd13.160221
16. Hu MD, Lawrence KG, Bodkin MR, Kwok RK, Engel LS, Sandler DP. Neighborhood Deprivation, Obesity, and Diabetes in Residents of the US Gulf Coast. *American Journal of Epidemiology*. 2020;190(2):295-304. doi:10.1093/aje/kwaa206
17. Dale AM, Fischl B, Sereno MI. Cortical Surface-Based Analysis: I. Segmentation and Surface Reconstruction. *NeuroImage*. 1999/02/01/ 1999;9(2):179-194. doi:https://doi.org/10.1006/nimg.1998.0395
18. Desikan RS, Ségonne F, Fischl B, et al. An automated labeling system for subdividing the human cerebral cortex on MRI scans into gyral based regions of interest. *NeuroImage*. 2006/07/01/ 2006;31(3):968-980. doi:https://doi.org/10.1016/j.neuroimage.2006.01.021
19. Hamaker EL, Kuiper RM, Grasman RP. A critique of the cross-lagged panel model. *Psychol Methods*. Mar 2015;20(1):102-16. doi:10.1037/a0038889
20. McArdle JJ. Latent variable modeling of differences and changes with longitudinal data. *Annu Rev Psychol*. 2009;60:577-605. doi:10.1146/annurev.psych.60.110707.163612
21. Mund M, Nestler S. Beyond the Cross-Lagged Panel Model: Next-generation statistical tools for analyzing interdependencies across the life course. *Advances in Life Course Research*. 2019/09/01/ 2019;41:100249. doi:https://doi.org/10.1016/j.alcr.2018.10.002
22. Orth U, Clark DA, Donnellan MB, Robins RW. Testing prospective effects in longitudinal research: Comparing seven competing cross-lagged models. *J Pers Soc Psychol*. Apr 2021;120(4):1013-1034. doi:10.1037/pspp0000358

© 2023 Sakib MN et al. *JAMA Network Open.*
