# Supplementary material for: Bidirectional Associations Between Adiposity and Cognitive Function and Mediation by Brain Morphology in the ABCD Study
Source: JAMA Netw Open. 2023 Feb 16;6(2):e2255631. doi: 10.1001/jamanetworkopen.2022.55631 (PMC9936350; doi:10.1001/jamanetworkopen.2022.55631)
Supplement: Supplement 2. — Data Sharing Statement [file jamanetwopen-e2255631-s002.pdf]

Data Sharing Statement

Sakib. Bidirectional Associations Between Adiposity and Cognitive Function and Mediation by Brain Morphology in the ABCD Study. *JAMA Netw Open*. Published February 16, 2023. doi:10.1001/jamanetworkopen.2022.55631

Data
Data available: No

Additional Information
Explanation for why data not available: The data used for the analyses presented in this paper are from the Adolescent Brain Cognitive Development (ABCD) Study [https://abcdstudy.org; NIMH Data Archive (NDA)]. Data can be accessed by directly applying to the NDA.
